# A Mixed Methods Approach to Evaluate Partnerships and Implementation of the Massachusetts Prevention and Wellness Trust Fund

**DOI:** 10.3389/fpubh.2018.00150

**Published:** 2018-06-05

**Authors:** Rebekka M. Lee, Shoba Ramanadhan, Gina R. Kruse, Charles Deutsch

**Affiliations:** ^1^Clinical and Translational Science Center, Harvard Medical School, Boston, MA, United States; ^2^Prevention Research Center, Harvard T.H. Chan School of Public Health, Boston, MA, United States; ^3^Center for Community-Based Research, Dana Farber Cancer Institute, Boston, MA, United States; ^4^Division of General Internal Medicine, Massachusetts General Hospital, Boston, MA, United States

**Keywords:** implementation science, mixed methods research, asthma, hypertension, falls, tobacco

## Abstract

**Background:** Strong partnerships are critical to integrate evidence-based prevention interventions within clinical and community-based settings, offering multilevel and sustainable solutions to complex health issues. As part of Massachusetts' 2012 health reform, The Prevention and Wellness Trust Fund (PWTF) funded nine local partnerships throughout the state to address hypertension, pediatric asthma, falls among older adults, and tobacco use. The initiative was designed to improve health outcomes through prevention and disease management strategies and reduce healthcare costs.

**Purpose:** Describe the mixed-methods study design for investigating PWTF implementation.

**Methods:** The Consolidated Framework for Implementation Research guided the development of this evaluation. First, the study team conducted semi-structured qualitative interviews with leaders from each of nine partnerships to document partnership development and function, intervention adaptation and delivery, and the influence of contextual factors on implementation. The interview findings were used to develop a quantitative survey to assess the implementation experiences of 172 staff from clinical and community-based settings and a social network analysis to assess changes in the relationships among 72 PWTF partner organizations. The quantitative survey data on ratings of perceived implementation success were used to purposively select 24 staff for interviews to explore the most successful experiences of implementing evidence-based interventions for each of the four conditions.

**Conclusions:** This mixed-methods approach for evaluation of implementation of evidence-based prevention interventions by PWTF partnerships can help decision-makers set future priorities for implementing and assessing clinical-community partnerships focused on prevention.

## Introduction

The delivery of preventive services in community-based and clinical settings has tremendous potential to improve population health. However, these community and clinic-based preventive activities are rarely coordinated (1), even with evidence that clinical-community partnerships can improve health outcomes including smoking abstinence, perceived physical health, cholesterol levels and hypertension (2, 3). The potential of community-clinical partnerships to improve health is further emphasized by the finding that neighborhood or community-level determinants of health also impact the way patients interact with the healthcare system as measured by hospital readmissions (4) and emergency room visits (5). As healthcare systems become increasingly accountable for improving the health of populations, strategies for linking clinical systems and community-based partners are becoming essential (6).

Clinical-community collaborations offer an opportunity to create multi-level, sustainable change. Thousands of coalitions, alliances, and other forms of inter-organizational health focused partnerships were formed over the past two decades (7–9). These intersectoral partnerships are critical for addressing complex public health challenges. They can marshal complementary human and social capital, embed interventions in the broader public health system, and offer opportunities to address problems that cannot be solved by an organization or sector in isolation (8–11). Although collaboration across sectors or institution types is not without its challenges (12), coalitions and intersectoral partnerships have successfully impacted health disparities broadly (13), as well as in improved diabetes, HIV/AIDS, and substance abuse outcomes (14–16).

The clinical-community partnerships in this project implemented evidence-based interventions that address hypertension, pediatric asthma, falls among older adults, and tobacco use throughout Massachusetts. In 2012, as the second stage in Massachusetts' ground-breaking health reform initiative, the legislature passed Massachusetts General Law Chapter 224 (17). Among other things, it established the Prevention and Wellness Trust Fund (PWTF), which provided more than $42 million over 4 years to nine community-clinical partnerships. The Massachusetts Department of Public Health led the initiative, competitively selecting nine partnerships in diverse communities across the state and providing technical assistance to implement specified evidence-based interventions. The conditions and interventions were chosen for implementation because they were determined to be more likely than others to show changes in outcomes and costs, and positive return on investment, in the span of 3 years. The nine chosen communities exceeded state-wide prevalence of the priority conditions, were more racially and ethnically mixed, and had higher rates of poverty than the state average (18). The funded partnerships varied in configuration and ranged in size from 40,000 to 140,000 people; some were single cities, others included multiple cities and towns, and one constituted an entire county. Fifteen percent of the state population resides within the nine funded partnerships. All partnerships included a city/regional planning agency, a clinical health provider, and a community-based organization. Their size range from 6 to 15 participating organizations. More details on the PWTF partnerships, decisions, interventions, and model are available in the project final report (19).

The initiative began in 2014 with a 6–9 month planning stage focused on capacity building. Communities developed partnerships among clinical providers and community-based organizations that linked and coordinated clinical and community-based strategies. The request for response specified that at least one intervention must involve bi-directional referrals from clinical to community organizations with feedback loops. For example, a community health center might partner with the YMCA to develop a system in which patients screened as hypertensive or at risk for falls are referred to community programming, and conversely YMCA members who express needs for clinical services are referred to the community health center. For most of the partnerships, full implementation began early in 2015. Table 1 lists the clinical and community evidence-based interventions for each health condition. Of the nine partnerships, all selected hypertension, eight selected falls among older adults, five selected tobacco cessation, and six chose pediatric asthma. MDPH provided grantee support, such as individualized technical assistance in evidence-based interventions, learning sessions to facilitate knowledge development and sharing across all grantees, and quality improvement evaluation. Partnerships were encouraged to culturally adapt interventions to meet the needs of their local communities.

**Table 1:** Clinical and community interventions implemented as part of the Prevention and Wellness Trust Fund.

**Intervention**	**Tier**	**Partnerships**
**HYPERTENSION**		
Evidence-Based Guidelines for Hypertension Screening	Clinical 1	9
Chronic Disease Self-Management Programs	Community 1	8
Self-Monitored Blood Pressure with Additional Support	Community 2	6
Diabetes Prevention Program for Patients with Hypertension and Pre-Diabetes	Community 2	3
**FALLS AMONG OLDER ADULTS**		
Stopping Elderly Deaths, Accidents, and Injuries: Clinical Risk Assessment	Clinical 1	8
Assisted Home Safety Assessment	Community 2	8
Matter of Balance	Community 2	8
Tai Chi	Community 2	6
**TOBACCO CESSATION**		
US Preventive Services Task Force Screening Guidelines	Clinical 1	5
Tobacco Cessation Counseling	Community 1	5
Promoting Smoke-Free Environments	Community 2	5
**PEDIATRIC ASTHMA**		
Care Management for High Risk Asthma Patients	Clinical 1	6
Asthma Self-Management in Primary Care	Clinical 2	4
Home-Based Multi-Trigger Multi-Component Intervention	Community 1	5
Comprehensive School-Based Asthma Programs	Community 2	4
Comprehensive Head Start-Based Asthma Programs	Community 2	2

The communities were required to jointly fund a rigorous independent evaluation of the PWTF to determine if it met its explicit legislative objectives: (1) a reduction in the prevalence of preventable health conditions; (2) a reduction in health care costs or the growth in health care cost trends associated with these conditions; and (3) an assessment of which populations benefited from any reduction. While not specified in the authorizing legislation, the Prevention and Wellness Advisory Board (PWAB) created by Chapter 224 strongly recommended the additional systematic collection of data that illustrate the implementation experiences in PWTF communities.

The purpose of this paper is to present a mixed methods approach to assess the PWTF implementation experience. While an outcome evaluation is critical to establishing success, embedding quantitative surveys and qualitative interviews that assess how these partnerships function and what contextual factors influence implementation will help to provide actionable findings. This paper draws upon implementation science, social network analysis, and a mixed methods design to understand these complexities.

First, the field of dissemination and implementation science is concerned with generating knowledge beyond clinical trials and effectiveness research to investigate change in real-world settings. In this study, we define implementation “as the way and degree to which an intervention is put into place in a given setting” (20). Fundamental to implementation science is the concept of integrating evidence-based interventions within a community or clinical setting and creating partnerships and supportive delivery systems to support the use of evidence-based interventions. At the core of this science is inquiry into the contextual factors that influence successful implementation of evidence-based interventions. To ground our inquiry, we applied the Consolidated Framework for Implementation Research (CFIR), an established framework that supports identification of actionable factors that influence success within five domains: the inner setting, the outer settings, characteristics of individuals, characteristics of the intervention, and processes (21).

Next, it was important to examine the composition, structure, and functions of the PWTF partnerships in the context of implementing evidence-based interventions. Social network analysis is a natural fit for evaluation of the function and impact of community-clinical partnerships, as it focuses on relationships (here, between organizations) and takes a systems perspective (22). Social network analysis has been applied effectively to the study of a range of collaborative efforts among organizations engaged in health promotion activities (23–25). Using the methods of social network analysis, it becomes possible to assess the form and function of a network, identify key actors and the types of resources exchanged across the network, assess the sustainability and strength of relationships, assess opportunities to strengthen the network's impact on a set of health outcomes, and assess challenges or drawbacks to collaboration (11). In this way, social network analysis affords the opportunity to explore the ways in which community-clinical partnership networks can be utilized to create change and achieve intended implementation outcomes (and ultimately, intended health outcomes) in the organizations and communities of interest.

**Figure 1:**
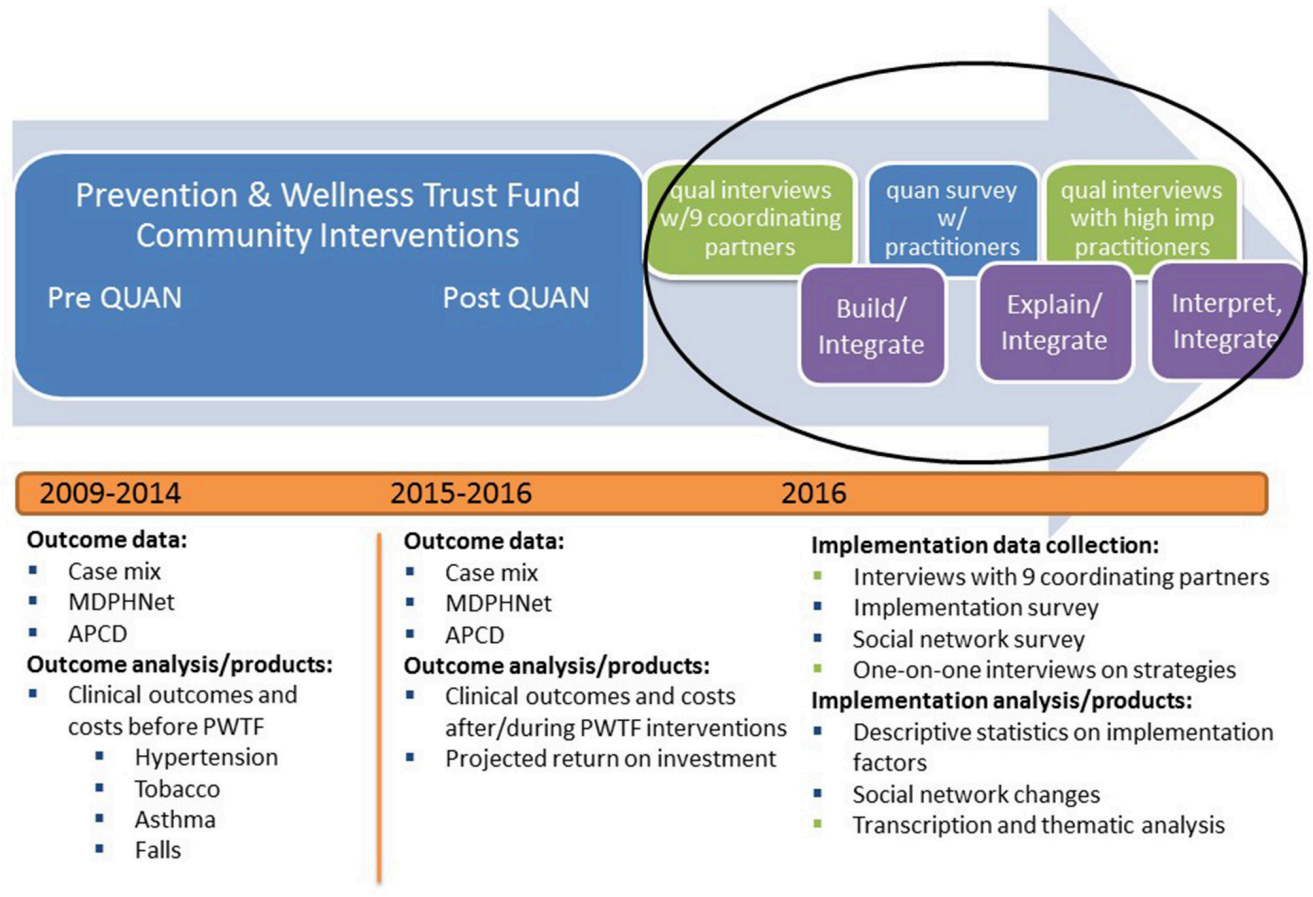
Multi-phase explanatory sequential mixed methods design embedded in the Prevention and Wellness Trust Fund Evaluation.

Finally, mixed methods research is the collection and analysis of quantitative and qualitative data, which is often employed to understand complex research problems for which one methodology is not sufficient (26). Mixed methods studies must use rigorous quantitative and qualitative methods and explicitly integrate or link these two types of data for a more comprehensive investigation of the topic at hand (26). Using mixed methods can be helpful for understanding the perceptions of practitioners and end-users of a given evidence-based intervention (27). A mixed methods design also aligns well with the need to conduct multi-level assessments of implementation efforts (e.g., collecting data at the community, clinic, provider, and patient levels) (28, 29). In this study, we use a multi-phase, explanatory sequential mixed methods design embedded in a large evaluation project to gain a more comprehensive understanding of implementation of the Prevention and Wellness Trust Fund interventions (Figure 1) (26, 30). Building three rapid phases of data collection and analysis upon one another is intended to explain what success looks like in this state-wide implementation of clinical-community linkages to build population-level disease prevention and management systems.

This mixed methods external evaluation will be useful to a variety of stakeholders, including legislators and other policymakers who need to know what PWTF accomplished and what next steps are indicated; implementing communities and agencies who need to know what worked and what didn't, and for whom; and other communities that want to learn from the PWTF experience.

## Materials and methods

**Figure 2:**
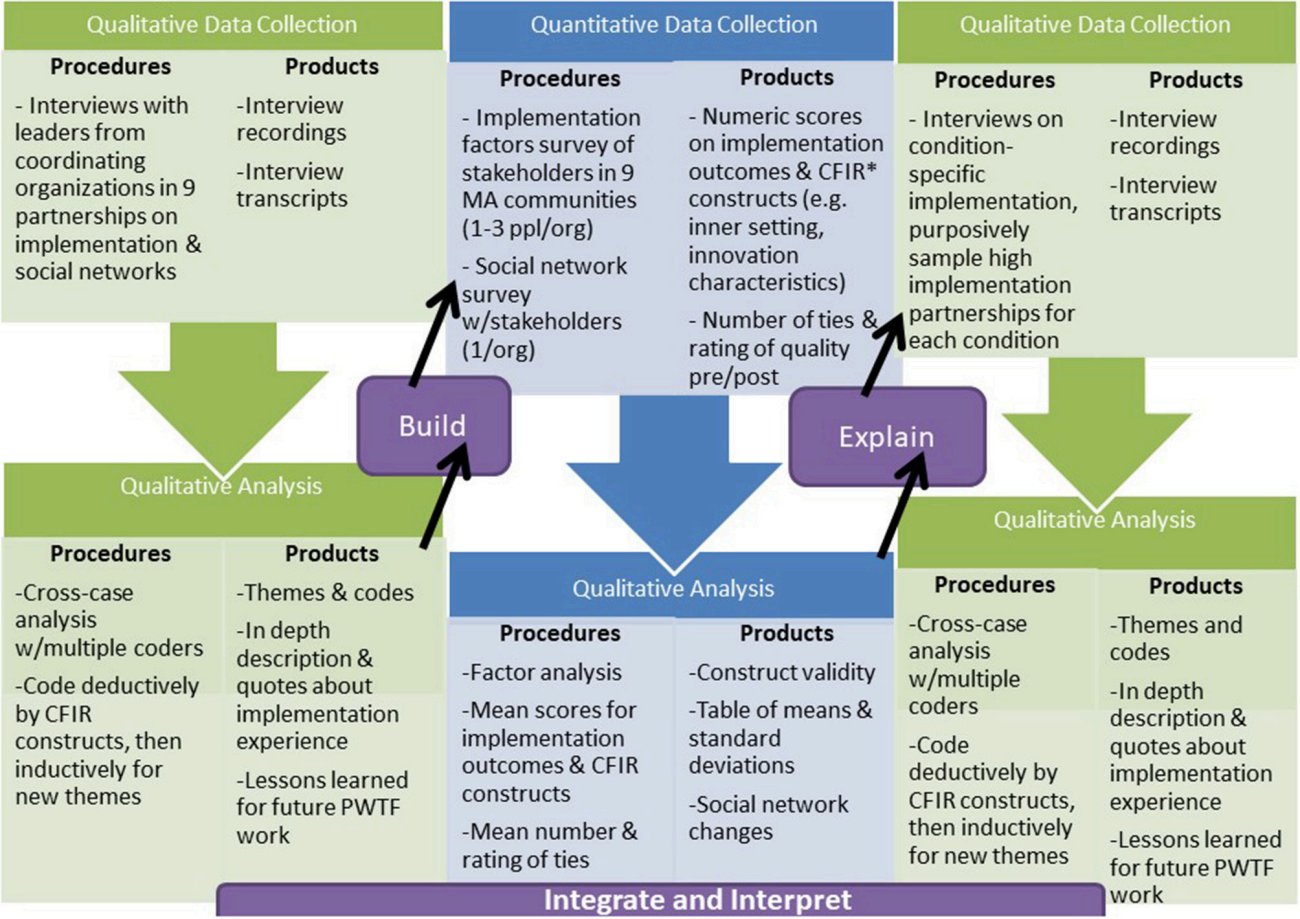
Step-by-step protocol for the multi-phase, explanatory mixed methods design for the Prevention and Wellness Trust Fund implementation evaluation.

We used a multi-phase explanatory mixed methods design embedded in a larger evaluation to investigate what interventions work for whom and in what settings—key issues at the core of implementation science (see Figure 1). First, we conducted semi-structured qualitative telephone interviews (lasting about 1.5 h) with at least two leaders from each of the nine partnerships. Key informant interviews are in-depth discussions that offer insight into participants' perceptions and opinions and are suited for exploratory research (31). They are often conducted with an individual, but we chose to conduct them with leadership teams to gather high-level perspective and a sense of daily implementation efforts. The interview findings were used to develop a quantitative survey to assess the implementation experiences of 172 staff from participating clinical and community-based organizations and a social network analysis to assess changes in the relationships among 70 PWTF organizations. The quantitative survey data on ratings of perceived implementation success were used to purposively select 24 staff for interviews. These 1.5-h interviews (in person whenever possible) were intended to explore the most successful experiences of implementing evidence-based hypertension, falls, tobacco, and asthma interventions. We chose interviews at this stage rather than staff focus groups because we sampled different cadres of staff (e.g., physicians, partnership coordinators, community health workers). We expected some staff would be more comfortable describing challenges or barriers to implementation in one-on-one interviews versus focus groups which may have included more senior staff and leaders from their communities. Detailed descriptions of each of the phases of the mixed methods implementation evaluation are below and described visually in Figures 1, 2 with details on the project timeline, data collection and analyses activities, and products. The Consolidated Framework for Implementation Research (CFIR) guided the development of this evaluation (21). The Harvard Office of Human Research Administration (IRB) determined that full review and approval was not required for this study. It has been approved by the Office of Human Research Administration staff and the proposal was reviewed by the Department of Public Health's Institutional Review Board.

### Phase 1: qualitative interviews with coordinating partners

In March 2016, key informant interviews in Phase 1 served as an initial, high-level qualitative exploration of the implementation experience in each partnership and helped to adapt existing survey items to identify contextual influences on PWTF implementation in Phase 2.

#### Sampling, recruitment, and administration

Each partnership had one organization that served as the coordinating partner, meaning that it was responsible for leading and managing the initiative. The Massachusetts Department of Public Health identified participants from the coordinating partners for the Phase 1 qualitative interviews. The 2–4 key informants from each community included the current PWTF project manager from each partnership, plus additional interviewees with a large breadth of knowledge about this project. Participants included health department directors, community health center senior leadership, healthcare system administrators, and past project managers in communities that had experienced leadership turnover. Prior to interviews, the study team emailed each PWTF project manager a one-page overview detailing the purpose and expectations of each phase of the implementation evaluation. All interviews were scheduled via email and conducted over the phone at the convenience of coordinating partners. The research team conducted 1.5-h telephone interviews with each coordinating partner team. All coordinating partners agreed to participate in Phase 1 interviews.

#### Measures

Implementation constructs explored in the Phase 1 interview included the implementation experience as well as an exploration of the contextual influences on implementation. To capture implementation experience, we included prompts related to buy-in among leadership and staff, details of intervention adaptation and delivery, the role of community health workers in supporting community-clinical partnerships to implement evidence-based interventions, and the connection between intervention implementation and health equity issues. The research team adapted an existing interview guide (32) based on the Consolidated Framework for Implementation Research (CFIR) to the PWTF settings and outcomes, attending to each of the five CFIR domains: inner setting (e.g., leadership engagement, resources) characteristics of the intervention (e.g., complexity, relative advantage), characteristics of individuals (e.g., role, turnover), outer setting (e.g., community context), and processes (e.g., planning, engaging champions) (21). The full interview guide is available in Supplementary Material 1 and example of qualitative interview questions appear in Table 2.

**Table 2:** Sample qualitative interview and quantitative survey questions aligned with the Consolidated Framework for Implementation Research (CFIR).

**Construct**	**Qualitative Interview Questions**	**Quantitative Survey Items**
**INNER SETTING**		
Leadership engagement	What level of involvement and support for the Prevention Wellness Trust Fund have you seen or heard from leaders within your institution during the implementation period?	The leadership makes sure that we have the time and space necessary to discuss changes to improve our practiceslatha *5-point Likert scale*
Available resources	What costs were incurred by implementing the Prevention Wellness Trust Fund initiative?latha *Probes for personnel time, training, purchase*	The following are available to make [insert evidence-based intervention] work in our partnerships: equipment and materials, sufficient staffing, data systems/IT supportlatha *5-point Likert scale*
**OUTER SETTING**		
External policies and incentives	Were there any concurrent initiatives that influenced your ability to implement the PWTF interventions? Examples include PCMH certification, transition to ACO model, EHR changes, behavioral health integration efforts Did other initiatives help you to implement PWTF activities? How? Did you delay or decline to do other initiatives because of the PWTF? What did you delay or decline?	Has your practice participated in any of the following initiatives or activities at the same time as the PWTF project activities? Patient Centered Medical Home certification Any electronic health record transition(s) New risk-sharing or accountable care organization contracts Meaningful Use attestation
**PROCESSES**		
Goals	To what extent has your organization set goals for implementing the intervention? Have these changed over time?	Organizational leaders establish clear goals for using [insert evidence-based intervention] to address [health condition]latha *5-point Likert scale*
**CHARACTERISTICS OF THE INTERVENTION**		
Complexity	How would you gauge the time and effort required to implement the Prevention Wellness Trust Fund over the course of the project?	Overall, I believe that is was complicated to implement [insert evidence-based intervention]latha *5-point Likert scale*
**CHARACTERISTICS OF THE INDIVIDUAL**		
Turnover	Did your organization experience any turnover this year? How did that influence your ability to implement Prevention Wellness Trust Fund evidence-based interventions?	Has your organization experience any turnover of staff working on PWTF since September 2014? If yes, how many staff have left?

The social network analysis portion of the interview guide examined two classes of networks: (a) intra-partnership networks (relationships between PWTF organizations within each of the nine partnerships) and (b) inter-partnership networks (relationships between the nine partnerships). For the intra-partnership network assessment, the first step was to define the set of organizations of interest; in this case, all organizations involved with PWTF implementation (33). For each partnership we used the list from the MDPH as a starting point and then reviewed it with partners to revise as needed. Second, the interview guide included prompts to define relationships of interest. The literature suggests that important relationships linked to creating practice change in healthcare settings include communication, collaboration or competition, exertion of influence, and exchanging resources (25, 34). We asked about these and also prompted respondents to identify other important interactions or exchanges that supported their PWTF goals. Finally, we asked a set of questions to explore the role of additional, unofficial partners in the PWTF initiative. For example, a given community-based organization may be the official delivery site for a given evidence-based intervention, but may link with other local organizations for recruitment or other activities.

For the inter-partnership assessment, the interview guide focused on relationships among the nine participating partnerships, as they had been brought together as part of a quality improvement learning collaborative to support PWTF goals. The interviews focused on the range of network relationships involved in implementing evidence-based interventions through the PWTF. We also asked about the range of benefits derived from engaging with other partnerships and expected sustainability of these relationships.

#### Data management and analysis

Interview recordings were transcribed verbatim. Data were managed and prepared for analysis using NVivo qualitative data analysis software Version 11 (QSR International Pty Ltd. 2012. Melbourne, Australia). The research team reviewed transcripts for key constructs to include in the Phase 2 quantitative implementation and social network surveys. We conducted a cross-case analysis that began deductively coding according to contextual factors from CFIR, and then inductively added codes for new patterns and themes. Rigor was ensured with analysis triangulation; all interviews were coded by two researchers to ensure multiple perspectives (35, 36). Interview data were integrated with the phase 2 survey and phase 3 interview data, looking for concordant and discordant results (26).

### Phase 2: quantitative surveys

During May and June 2016 in Phase 2 of this evaluation, we fielded two online surveys to quantitatively identify the contextual factors that influenced implementation of the evidence-based interventions and assess the social networks within and between each partnership. Both surveys helped to adapt an existing guide (32) for follow-up in-depth interviews in Phase 3.

#### Sampling, recruitment, and administration

The research team worked with the Massachusetts Department of Public Health and coordinating partners to generate a list of all organizations that were part of each partnership. Next, coordinating partners indicated the health conditions and evidence-based interventions associated with each organization and listed the names, roles/titles, and email addresses for 1–3 contacts at each organization who were involved with implementing the evidence-based interventions. They were asked to include clinical staff of varying levels (doctors, nurses, and medical assistants), practitioners in community-based settings, and community health workers. One week prior to launching the surveys, the study team emailed each PWTF project manager to disseminate a one-page overview detailing the broad content areas of focus on the survey. Project managers from each partnership shared the overview with participants.

Both surveys were conducted online via REDCap electronic data capture tools (37). The implementation survey was administered to all contacts identified by the coordinating partners (*N* = 2 14). The social network survey was administered to one representative at each organization designated as the lead for the PWTF (*N* = 90). Participants were invited to complete the surveys by email. They were given a 2-week window to respond to the surveys, with reminders sent at 1 week and 1 day before the official close. Coordinating partners assisted in encouraging survey participation. Participants were incentivized to complete the implementation survey with a chance to win a raffle for a $75 gift card. A total of 172 individuals completed the implementation survey (response rate = 80%) and 72 people completed the social network survey (response rate = 80%).

#### Measures

The research team adapted existing validated survey items (38, 39) to the PWTF settings and outcomes using findings gleaned from the Phase 1 interviews. Items assessed the perceived degree of implementation for each evidence-based intervention as well as contextual domains in the CFIR (21). A 4-point Likert scale captured the degree of implementation, with the following ratings: 0 (no implementation); 1 (“we are in the early stages of implementation”); 2 (“we have implemented this strategy, but inconsistently”); and 3 (“we have implemented this intervention fully and systematically”). The CFIR survey items were measured on a 5-point Likert scale with responses ranging from 1-strongly disagree to 5-strongly agree. We also included items to capture title, role, age, gender, race/ethnicity, education, language spoken, and years of experience. Adaptations to the survey were made based on qualitative data provided by the coordinating partners in Phase 1. For instance, sufficient staffing and data systems/IT support were frequently named as important resources influencing implementation; therefore, we created discrete items to assess these factors quantitatively on the survey. Using the qualitative data to adapt the quantitative survey ensured we could measure the frequency of these contextual influences in the large pool of 172 clinical and community-based implementers. The full survey is available in Supplementary Material 2 and Table 2 includes examples of survey items.

The quantitative, intra-partnership social network analysis utilized the list of organizations involved with PWTF implementation from the Phase 1 interviews and asked about relationships with all other members of the partnership. For example, if a given partnership included 7 organizations, we surveyed each organization about their relationships with the other 6 organizations. The social network analysis focused on a core set of relationships identified in Phase 1 as important for implementation: collaboration, sharing information/resources, sending referrals, receiving referrals, providing/receiving technical assistance or capacity-building, providing/receiving access to community members. We also asked questions about the sustainability of reported connections after funding is completed. Finally, we asked questions to prompt respondents to identify up to five additional partners involved in the execution of the evidence-based program or strategy. The quantitative, inter-partnership social network analysis included questions about relationships (using the same list provided above) with the other partnerships. Once more we asked about expected sustainability of connections after funding ends.

#### Data management and analysis

The research team analyzed quantitative survey data in SAS v9.4 (SAS Institute: Cary, NC). We calculated descriptive statistics (e.g., means of implementation outcomes and CFIR constructs) for all outcomes. A summary score for each evidence-based intervention was created for each partnership by averaging ratings of implementation from all respondents in each partnership. These 4-point scale summary scores were used to classify partnerships as “high implementation” using self-reported scores for each health condition. High implementation partnerships for each condition had summary scores for each evidence-based intervention that were higher than the PWTF average. Social network data were analyzed using a combination of the dedicated network analysis software UCINET (Analytic Technologies: Lexington, KY) and SAS v9.4. Quantitative social network analyses emphasized analysis of the relationships within the official set of network members for each partnership. The analyses linked social network metrics with implementation outcomes.

### Phase 3: qualitative interviews with implementers

In July and August 2016, the final phase of our evaluation, we conducted follow-up in-depth interviews with practitioners charged with implementation. The interviews focused on developing a more comprehensive understanding of the experience of implementing the evidence-based hypertension, falls, asthma, and tobacco interventions in real world clinical and community settings.

#### Sampling, recruitment, and administration

The research team sampled “high implementation” partnerships for participation in the Phase 3 interviews. The 4-point summary scores from Phase 2 surveys were used to classify partnerships as “high implementation” using self-reported scores for each health condition.

After high implementation partnerships were identified, the research team sampled 4–6 individuals (at least one clinical partner and one community partner) from each partnership for interviews. These individuals were purposively sampled from the list of implementation survey respondents in an effort to conduct information-rich interviews. For instance, Phase 3 interviews for falls among older adults in one partnership included speaking with a community health worker who conducted falls assessments and referrals within a community health center, a falls prevention coordinator from an elder services organization responsible for home safety assessments, folks leading Matter of Balance and Tai Chi classes at the YMCA and via city recreation, as well as the director of a local non-profit organization.

All 1.5-h interviews were scheduled via email and conducted in-person at the convenience of the participants whenever possible (two interviews were conducted over the phone). Interviews were audio recorded and transcribed verbatim. Participants were compensated with a $25 gift card. All people invited for Phase 3 interviews agreed to participate.

#### Measures

Similar to the Phase 1 formative interviews, the research team adapted an existing interview guide (32) based on the CFIR to the PWTF settings and outcomes. The adaptation included tailoring the interview to investigate findings from the quantitative surveys of Phase 2. Targeted probes for CFIR items with the highest or lowest average ratings on the survey were added to the interview. This was done to explore barriers and facilitators to implementation in greater depth. For example, respondents' extreme rating of the complexity of interventions and resources such as staffing led our team to add probes to the interview guide to gain a better understanding of what intervention complexity and staffing constraints looked like from the perspectives of those who were implementing the interventions in real world settings. Implementation constructs explored in the Phase 3 follow-up interview included the experience of implementing specific evidence-based interventions and an exploration of the contextual influences on implementation. Elements of the implementation experience include buy-in among leadership and staff, a description of how interventions were adapted and delivered, the role of community health workers, and strategies to address health equity. Clinical partners were also asked to discuss how quality of care initiatives impacted implementation of the PWTF interventions (40). All five CFIR domains were explored in this phase for each target health condition (21). The full interview guide appears in Supplementary Material 3 and there are examples of qualitative interview items in Table 2.

The analysis of Phase 2 network data highlighted the diversity of partnership structure for organizations working together to implement evidence-based interventions through the PWTF. We explored this further by asking implementers to describe their experiences with community-clinical linkages as part of the PWTF initiative. We also asked a series of questions about partnership sustainability to compare and contrast descriptions provided by implementers vs. descriptions provided by partnership leaders (Phase 1).

#### Data management and analysis

All interview audio-recordings were transcribed. Data were managed and prepared for analysis using NVivo qualitative data analysis software Version 11 (QSR International Pty Ltd. 2012. Melbourne, Australia). We conducted a cross-case analysis that began deductively coding according to contextual factors from CFIR, and then inductively added codes for new patterns and themes (35, 36). One-third (8 of 24) of transcripts were coded by a second researcher to build consensus around all codes and themes. Phase 3 interview data were integrated with survey and Phase 1 key informant interview data, looking for concordant and discordant results (26).

## Discussion

This paper describes the design of a mixed methods approach for evaluating the implementation of clinical-community partnerships through The Prevention and Wellness Trust Fund. This study design will help us gain a comprehensive understanding of this complex approach for engaging communities in implementing evidence-based interventions across Massachusetts. To create an evaluation protocol that was truly mixed-methods, rather than simply multi-method, it was critical to explicitly and strategically find points in the evaluation process to integrate our qualitative data (41). In our multi-phase, explanatory sequential mixed methods design embedded in the larger PWTF evaluation, data were integrated or linked in several ways. First, while the initial mandated evaluation focused solely on the analysis of large quantitative datasets of medical claims, hospital discharges, and aggregated electronic health records, the PWTF advisory board and our research study team also prioritized embedding qualitative data into the larger evaluation to understand the complexities of the local implementation experiences. We also integrated quantitative and qualitative data to build implementation survey measures. The initial interviews with key informants were used to prioritize and adapt survey items for a tailored quantitative assessment of partnership social networks and implementation of the PWTF evidence-based interventions with a broader sample of implementation stakeholders in phase 2. Additionally, the study followed up on surveys with a second round of interviews as a means of explaining the quantitative results in greater depth. In this explanatory process, we used quantitative data on perceived level of implementation to sample “high implementation” partnerships and create qualitative probes to examine contextual implementation factors that were quantitatively rated as influential. This complex design presented the challenge of multiple phases depending on the success of earlier phases and determining how much data is sufficient to move forward to each subsequent mixed methods phase. For example, deciding how much quantitative analysis of the online survey should be conducted to inform the sampling and adaptation of the qualitative follow-up interviews.

The Prevention and Wellness Trust Fund sought to build and use partnerships to implement complex interventions in complex systems (42), meaning that a group of connected, “un-siloed” interventions addressing four priority health conditions were implemented in coordination across a variety of settings (e.g., hospitals, community health centers, schools, YMCAs, housing). By measuring the function and impact of partnerships within and between communities implementing evidence-based prevention programs, this evaluation is designed to better understand how to set up and support community-based prevention efforts. Accountable Care Organizations, which strive to develop clinic-community partnerships to improve the health of populations may use PWTF as a prototype. Using implementation science, interviews and surveys may help identify best practices for tailoring evidence-based interventions to unique contexts and constituents. The mixed methods study design also allows us to detail the challenges of clinical-community linkages, which are vital both in the narrow sense of promoting the use of specific evidence-based programs or practices, but also in a broader sense of, supporting sustainable community-level, systems changes (43).

The use of a mixed methods approach to understanding the implementation of evidence-based practices in clinical-community partnerships draws on the strengths of both qualitative and quantitative methods, but it is not without limitations. First, time constraints presented challenges in several ways, given that the external evaluation was only funded for the second year of a three-year implementation period. Limited time meant that our study was only able to conduct in-depth follow-up interviews with people implementing the interventions in “high implementation” partnerships. If we had more time, we could have prioritized exploring the implementation process and contextual factors within partnerships that have less success in greater depth with follow-up interviews that could further our understanding of implementation challenges. Time also limited our ability to use more objective quantitative measures, such as program reach or changes in clinical outcomes, to sample “high implementation” partnerships. We were also limited in our ability to evaluate how partnerships were trained and subsequently implemented interventions to address health equity, with only one question on interviews directed toward this topic.

In sum, this paper details the research protocol for the external evaluation of the implementation of the Prevention and Wellness Trust Fund. Subsequent implementation research from this project aims to describe how the hypertension, falls, asthma, and tobacco evidence-based interventions were implemented and identify actionable contextual factors that influenced implementation in the nine partnerships. The mixed methods approach will provide data that appeals to a range of constituents—from scientists to policymakers to public health and clinical practitioners. The findings from this study will be valuable for understanding what PWTF has accomplished and to help other communities planning to set-up or support community-clinical partnerships to deliver evidence-based preventive services.

## Author contributions

RL served as the lead author on the paper, contributing to conceptualization, literature summary, development of data collection measures, drafting/editing all sections of the paper, tables, and figures. SR contributed to conceptualization of the paper, literature summary, development of data collection measures, and drafting/editing methods and discussion. GK contributed to conceptualization of the paper, development of data collection measures, and drafting/editing the introduction and methods. CD served as senior author on the paper, contributing to conceptualization of the paper, development of data collection measures, and drafting/editing the introduction and discussion.

### Conflict of interest statement

The authors declare that the research was conducted in the absence of any commercial or financial relationships that could be construed as a potential conflict of interest.
