# Supplementary material for: A Mixed Methods Approach to Evaluate Partnerships and Implementation of the Massachusetts Prevention and Wellness Trust Fund
Source: Front Public Health. 2018 Jun 5;6:150. doi: 10.3389/fpubh.2018.00150 (PMC5996756; doi:10.3389/fpubh.2018.00150)
Supplement: Supplementary file 1 [file Data_Sheet_1.DOC]

Hello, my name is [name of interviewer].

**Purpose of Interview:**

The purpose of this interview is to better understand the implementation experience of your Prevention Wellness Trust Fund partnership. This includes the experience of your partnership development as well as planning, developing systems, and delivering interventions. We are also interested in understanding the barriers and facilitators to implementation your partnership has faced during the Prevention Wellness Trust Fund initiative with the intent to gather lessons learned and improve future work in this area. Throughout the interview, I will refer to this work generally as the Prevention Wellness Trust Fund initiative. However, sometimes I will ask about your work around specific health conditions (e.g. asthma or hypertension).

This interview will help inform the development of a questionnaire that will allow us to learn more about the experiences of a large number of staff working in clinical settings and community-based organizations participating in the Prevention Wellness Trust Fund project. Later in the year, we will conduct one-on-one interviews with a sample of clinical staff, practitioners, and community health workers about implementation of specific interventions, such as the Matter of Balance program to address elder falls or home visits to address asthma.

**Logistics and Confidentiality:**

In terms of logistics, this interview will be audio taped so that we have an accurate record of your thoughts. Please be assured that the tapes and your transcript will be kept confidential, so please answer openly and honestly. If, at any time, you feel that the questions are too sensitive, I would be happy to turn off the recorder during that portion of questioning. You may also skip any questions you wish during the interview.

Do you have any questions for me? [Answer any questions]

Are you ready to begin? I’m going to start recording now.

**I. BACKGROUND** (30 Minutes w/ Introduction)

**1. First, to help me understand your role and who was involved with the Prevention Wellness Trust Fund initiative, will you please describe your role within your organization?** Background information

*PROBES*

- What is your title and role within your organization?
- Who do you report to?
- What is your role in the *Prevention Wellness Trust Fund initiative*?

**2. What other units/services/people WITHIN your organization did you work with to implement the Prevention Wellness Trust Fund initiative, and what was the role for each?** Networks & Communications – inside org

As you may have heard, we will be doing a social network analysis to understand the connections that make up the PWTF infrastructure. First, I would like to talk to you about the relationships between organizations in your PWTF partnership. We want to talk to all of the key players for each of your partnership’s selected health conditions.

**3. There are a number of partners listed as participating in the PWTF for your community. This is the list that was sent via email when we set up today’s call. I’d like to walk through each of these organizations briefly. Can you describe the role each of these partners play in the PWTF project?**

(Examples might include provide referrals, provide clinical interventions, provide programming, provide TA)

Networks & Communications –intra partnership/between orgs

**4. Are there other organizations that are not listed, but play a part in the PWTF? If yes, can you name these organizations for me and describe the roles they play?** Networks & Communications

**5. How did the organizations you’ve described come to be a part of the PWTF partnership?**

*PROBES*: Networks & Communications

- What kind of history do you have working with these organizations?
- Can you describe the partnerships you had prior to PWTF vs. new partnerships that have developed as part of this project?
- How did your partnerships determine which health topics conditions [elder falls/tobacco use/hypertension/pediatric asthma] to focus on in the Prevention Wellness Trust Fund project? Can you describe other reasons the led to your choice of health topics?

**6. What do partner organizations do to collaborate and meet PWTF goals?** Networks & Communications

*PROBES:*

- Do they share information / data? Share resources? Send / receive referrals? Are there any other important aspects of collaboration for PWTF goals?
- Can you help us understand how the list of partners in your community might be different for [asthma] vs. [falls] vs. [hypertension] vs. [tobacco control]?

**7. It’s been really helpful to understand the partnerships in effect at this time. Now, I would like you to think about these partnerships in the future, after the current PWTF funding cycle has ended. Do you expect these partnerships to be useful for sustaining the work on the selected health conditions after the initiative is completed? Why or why not? What about partnerships for purposes beyond these selected conditions?**

For those who said **yes**: What supports will be required to maintain these connections after the grant? What barriers could impact sustainability of these relationships? Networks & Communications

Now we will switch gears and talk about relationships among the nine partnerships participating in the PWTF.

**8. Has your partnership connected with other partnerships involved in the PWTF?**

If **yes**, which partnerships have you connected with? For what purposes? Networks & Communications –inter partnership

PROBE: What were some of the reasons you connected with other partnerships (Examples may include: sharing information, resources, sending / receiving referrals)

**9. Why did you connect with these vs. other partnerships?**

PROBE on whether it was characteristics of the other partnership leaders or communities, shared topical focus, history of previous collaboration, etc. Networks & Communications –inter partnership

**10. Once more, if you think about the time after the current PWTF funding cycle has ended, would these connections be useful for continuing the work on the selected health conditions after the initiative ends? Why or why not? What about partnerships for other purposes beyond these selected conditions?**

For those who said **yes**: what supports will be required to maintain these connections after the grant? What barriers could impact sustainability of these relationships?

**11. Prior to this initiative, what kind of strategies, if any, were you already using to address tobacco use?**

PROBE *about topic areas of focus for the community*

- **What about to address hypertension?** Relative Advantage
- **What about to address youth asthma?** Relative Advantage
- **What about to address elder falls?** Relative Advantage

**II. IMPLEMENTATION EXPERIENCE** (30 Minutes)

Now, I would like to hear about the process you went through to implement changes through the Prevention Wellness Trust Fund Project. [Refer to community work plan]. When I refer to implementation I mean the process of putting to use or integrating evidence-based interventions within a community or clinical setting—this could include intervention delivery as well as developing supportive systems.

Let’s start by talking about [elder falls/tobacco use/hypertension/pediatric asthma].*Repeat 1 & 2 for each condition.*

**1. In general, can you explain how buy-in for the initiative occurred? Among leadership? Among staff?**

**2. Now, thinking about the whole process involved with addressing [elder falls/tobacco use/hypertension/pediatric asthma], can you please tell me how the evidence-based interventions were implemented in your community.**

PROBES:

- What were the easiest changes to implement?
- What were the most challenging changes to implement?
- How have clinical and community organizations worked together? *This was often already answered*
- How have community health workers contributed to these changes?
- How have you worked to address health equity to your implementation of these changes?
- Have you made any adaptations to the evidence-based interventions? If so, please describe.

**3. In thinking about the entire Prevention Wellness Trust Fund project, what has not been accomplished that was part of the plan? What happened?**

Now, I am interested in learning more about your thoughts on the types of factors (both positive and negative) that have affected your ability to implement evidence-based interventions through the Prevention Wellness Trust Fund project.

**4. Very broadly, can you describe the major factors that you think influenced your ability to implement the Prevention Wellness Trust Fund project?**

*PROBES*

- Characteristics of the intervention/approach (such as the compatibility of the DPH-led TA and action planning with the way you do work in your organization and the complexity of the evidence-based strategy)
- Organizational infrastructure (e.g., available resources, size, physical layout, internal policies, leadership)
- Characteristics of individuals within the organization (e.g., role and experience of those involved)
- Influences outside of the organization (e.g., Local, state, or national policies; community characteristics; income, race, ethnicity of population served).

**5. Did these factors that influenced implementation differ by health condition?**

*PROBES*

- Were there barriers or facilitators specific to your work involving tobacco use?
  - hypertension?
  - pediatric asthma?
  - elder falls?

**III. SUPPORT & ENGAGEMENT** (5-10 Minutes)

Now l want to hear briefly about any support and engagement you may have received along the way.

**1. What is your perception of the quality of the technical assistance, supporting materials, packaging, and bundling of the Prevention Wellness Trust Fund evidence-based interventions?** Design Quality & Packaging

*PROBES*

- Learning collaboratives?
- TA calls/meetings with MDPH staff?
- Others?

**2. What level of involvement and support for the Prevention Wellness Trust Fund have you seen or heard from leaders within your institution during the implementation period?** Readiness for Implementation – Leadership Engagement

*PROBES*

- What are the roles of the leaders that have been most involved?
- How did their level of support or involvement differ by health condition?
- How were they involved or updated about the changes you planned/implemented?

**3. Did any vocal staff, patients, elected officials, or community members emerge that either helped facilitate or inhibit this initiative? What happened?** Champions…Opinion Leaders

**IV. OPERATIONAL EXPERIENCES** (5-10 Minutes)

Now that we have discussed what you have been doing as part of the Prevention Wellness Trust Fund initiative, we want to know more about what it took to complete these changes.

**1. How would you gauge the time and effort required to implement the Prevention Wellness Trust Fund over the course of the project?** Complexity

*PROBES*

- Of the organizations/people involved in the work, which did you find to be most critical to your efforts?
- Did the amount of time/effort differ by health condition?
- Was additional or unforeseen time or effort incurred in planning/getting buy-in? implementing the evidence-based interventions? collecting data?

**2. What costs were incurred by implementing the Prevention Wellness Trust Fund initiative?** Readiness for implementation - Available Resources

*PROBES*

- Personnel time?
- Training?
- New purchases?

**3. Did your organization experience any turnover this year? How did that influence your ability to implement Prevention Wellness Trust Fund evidence-based interventions?** Individual Stage of Change

**V. REFLECTIONS ON INITIATIVE** (5-10 Minutes)

Finally, we have a few questions to capture some of your overall reflections of this initiative.

**1. Based on your experience, are there any things you would change about the TA process to make it more helpful for implementation?** Process/Planning

*PROBES*

- Should certain individuals/units been engaged earlier?
- Were there unanticipated challenges that could have been considered more in planning?

**2. If you had the option, would you recommend continuing the Prevention Wellness Trust Fund initiative? Why or not?**

*PROBES*

- What would it take to keep this initiative going in your community?

**3. Do you have any specific suggestions for other organizations that have not yet started implementing the evidence-based interventions that are part of the Prevention Wellness Trust Fund initiative?**

*PROBE on coalition building & evidence-based interventions*

Thank you again for your time today and for all the work you have done on behalf of the Prevention Wellness Trust Fund initiative. We look forward to hearing about continued work being done.
