# Supplementary material for: A Mixed Methods Approach to Evaluate Partnerships and Implementation of the Massachusetts Prevention and Wellness Trust Fund
Source: Front Public Health. 2018 Jun 5;6:150. doi: 10.3389/fpubh.2018.00150 (PMC5996756; doi:10.3389/fpubh.2018.00150)
Supplement: Supplementary file 2 [file Data_Sheet_2.pdf]

PWTF Implementation Questionnaire - Spring 2016

Key: Consolidated Framework for Implementation Research (CFIR) contructs

For the purposes of disseminating this survey, items include alpha-numeric superscript to identify the corresponding CFIR construct (these do not appear in the actual survey). The survey uses the same subset of items for each health condition and intervention, therefore identifiers were inserted for the tobacco interventions only.

A. intervention characteristics
   1. relative advantage
   2. complexity
B. inner setting
   1. implementation climate
      a. compatibility
      b. relative priority
      c. goals & feedback
      d. learning climate
   2. readiness for implementation
      a. leadership engagement
      b. available resources
C. process
   1. executing
   2. reflecting & evaluating

Please select the answer that describes how much you agree or disagree with the following statements about your organization. These are general perceptions, not necessarily related to the PWTF.

We regularly take time to consider ways to improve how we do things.[B1d]

○ Strongly Disagree ○ Disagree ○ Neutral ○ Agree ○ Strongly Agree

People in our organization actively seek new ways to improve how we do things.[B1d]

○ Strongly Disagree ○ Disagree ○ Neutral ○ Agree ○ Strongly Agree

This organization encourages everyone to share their ideas.[B1d]

○ Strongly Disagree ○ Disagree ○ Neutral ○ Agree ○ Strongly Agree

The leadership makes sure that we have the time and space necessary to discuss changes to improve our practices.[B2a]

○ Strongly Disagree ○ Disagree ○ Neutral ○ Agree ○ Strongly Agree

Leadership in this organization creates an environment where things can be accomplished.[B2a]

○ Strongly Disagree ○ Disagree ○ Neutral ○ Agree ○ Strongly Agree

Organizational leadership promotes an environment that is an enjoyable place to work.[B2a]

○ Strongly Disagree ○ Disagree ○ Neutral ○ Agree ○ Strongly Agree

Organizational leadership strongly supports change efforts.[B2a]

○ Strongly Disagree ○ Disagree ○ Neutral ○ Agree ○ Strongly Agree

This organization learns from its mistakes.[B1d]

○ Strongly Disagree ○ Disagree ○ Neutral ○ Agree ○ Strongly Agree

When we experience a problem in the organization, we make a serious effort to figure out what's really going on.[B1d]

○ Strongly Disagree ○ Disagree ○ Neutral ○ Agree ○ Strongly Agree

We use data to guide our organization's operations.[C2]

○ Strongly Disagree ○ Disagree ○ Neutral ○ Agree ○ Strongly Agree

In general, when there is agreement that change needs to happen in the organization we have the necessary support in terms of:

	Strongly Disagree	Disagree	Neutral	Agree	Strongly Agree
budget or financial resources [B2b]	◯	◯	◯	◯	◯
training [B2b]	◯	◯	◯	◯	◯
staffing [B2b]	◯	◯	◯	◯	◯

Our organization consistently implements programs that are aligned with our mission and strategic plan. [C1]

◯ Strongly Disagree ◯ Disagree ◯ Neutral ◯ Agree ◯ Strongly Agree

Throughout the organization there is frequent and good communication about how different changes are going. [C2]

◯ Strongly Disagree ◯ Disagree ◯ Neutral ◯ Agree ◯ Strongly Agree

Tobacco

Did your partnership set goals around tobacco use through the PWTF? B1c

◯ Yes ◯ No

We are interested in YOUR involvement with the following best practices interventions for PWTF. Involvement could include coordinating & supporting implementation, making referrals, delivering interventions, conducting outreach, providing technical assistance, or supplying a venue/space.

Is your partnership implementing U.S. Preventive Services Task Force (USPSTF) screening guidelines to address tobacco use?

◯ Our partnership is working on this intervention, but I am not involved in implementation.
◯ No, our partnership is not implementing this intervention.
◯ Yes, we are in the early stage of implementation.
◯ Yes, we have implemented this strategy, but inconsistently.
◯ Yes, we have implemented this intervention fully and systematically.
◯ I don't know.

Is your partnership implementing promoting smoke-free environments to address tobacco use?

◯ Our partnership is working on this intervention, but I am not involved in implementation.
◯ No, our partnership is not implementing this intervention.
◯ Yes, we are in the early stage of implementation.
◯ Yes, we have implemented this strategy, but inconsistently.
◯ Yes, we have implemented this intervention fully and systematically.
◯ I don't know.

Is your partnership implementing tobacco cessation counseling?

◯ Our partnership is working on this intervention, but I am not involved in implementation.
◯ No, our partnership is not implementing this intervention.
◯ Yes, we are in the early stage of implementation.
◯ Yes, we have implemented this strategy, but inconsistently.
◯ Yes, we have implemented this intervention fully and systematically.
◯ I don't know.

Please select the answer that describes how much you agree or disagree with the following statements. We are interested in your experience implementing tobacco strategies, so there are no right or wrong answers to these questions.

Using USPSTF screening guidelines to reduce tobacco use is a top priority of the organization. B1b

◯ Strongly Disagree ◯ Disagree ◯ Neutral ◯ Agree ◯ Strongly Agree

Using USPSTF screening guidelines to reduce tobacco use is compatible with current activities in the organization. B1a

◯ Strongly Disagree ◯ Disagree ◯ Neutral ◯ Agree ◯ Strongly Agree

I think that using USPSTF screening guidelines to reduce tobacco use fits well with the way I like to work. B1a

◯ Strongly Disagree ◯ Disagree ◯ Neutral ◯ Agree ◯ Strongly Agree

Organization leaders establish clear goals for using USPSTF screening guidelines to reduce tobacco use. [B1c]

◯ Strongly Disagree ◯ Disagree ◯ Neutral ◯ Agree ◯ Strongly Agree

Organization leaders hold staff members accountable for achieving results of USPSTF screening guidelines. [B1c]

◯ Strongly Disagree ◯ Disagree ◯ Neutral ◯ Agree ◯ Strongly Agree

Organization staff are expected to help meet goals related to USPSTF screening guidelines. [B1c]

◯ Strongly Disagree ◯ Disagree ◯ Neutral ◯ Agree ◯ Strongly Agree

Organization staff get the support they need to implement USPSTF screening guidelines. [B1]

◯ Strongly Disagree ◯ Disagree ◯ Neutral ◯ Agree ◯ Strongly Agree

Organization staff get recognition for implementing USPSTF screening guidelines to reduce tobacco use. [B1]

◯ Strongly Disagree ◯ Disagree ◯ Neutral ◯ Agree ◯ Strongly Agree

The following are available to make USPSTF screening guidelines work in our partnership:

	Strongly Disagree	Disagree	Neutral	Agree	Strongly Agree
equipment and materials B2b	◯	◯	◯	◯	◯
public awareness/need B2b	◯	◯	◯	◯	◯
staff buy-in or support B2b	◯	◯	◯	◯	◯
sufficient staffing B2b	◯	◯	◯	◯	◯
data systems/IT support B2b	◯	◯	◯	◯	◯

Using USPSTF screening guidelines is more effective than our prior practices for reducing tobacco use. A1

◯ Strongly Disagree ◯ Disagree ◯ Neutral ◯ Agree ◯ Strongly Agree

It is difficult to train staff to implement USPSTF screening guidelines. A2

◯ Strongly Disagree ◯ Disagree ◯ Neutral ◯ Agree ◯ Strongly Agree

Overall, I believe that it was complicated to implement USPSTF screening guidelines. A2

◯ Strongly Disagree ◯ Disagree ◯ Neutral ◯ Agree ◯ Strongly Agree

I believe that using USPSTF screening guidelines has required my organization to make substantial changes to our previous practice. A2

◯ Strongly Disagree ◯ Disagree ◯ Neutral ◯ Agree ◯ Strongly Agree

USPSTF screening guidelines have required more work than can be done with current funding. A2

◯ Strongly Disagree ◯ Disagree ◯ Neutral ◯ Agree ◯ Strongly Agree

Promoting smoke-free environments to reduce tobacco use is a top priority of the organization. [B1b]

◯ Strongly Disagree ◯ Disagree ◯ Neutral ◯ Agree ◯ Strongly Agree

Promoting smoke-free environments to reduce tobacco use is compatible with current activities in the organization. [B1a]

◯ Strongly Disagree ◯ Disagree ◯ Neutral ◯ Agree ◯ Strongly Agree

I think that promoting smoke-free environments to reduce tobacco use fits well with the way I like to work. [B1a]

◯ Strongly Disagree ◯ Disagree ◯ Neutral ◯ Agree ◯ Strongly Agree

Organization leaders establish clear goals for using smoke-free environment promotion to reduce tobacco use. [B1c]

◯ Strongly Disagree ◯ Disagree ◯ Neutral ◯ Agree ◯ Strongly Agree

Organization leaders hold staff members accountable for achieving results of smoke-free environment promotion. [B1c]

◯ Strongly Disagree ◯ Disagree ◯ Neutral ◯ Agree ◯ Strongly Agree

Organization staff are expected to help meet goals related to smoke-free environment promotion. [B1c]

◯ Strongly Disagree ◯ Disagree ◯ Neutral ◯ Agree ◯ Strongly Agree

Organization staff get the support they need to implement smoke-free environment promotion. [B1]

◯ Strongly Disagree ◯ Disagree ◯ Neutral ◯ Agree ◯ Strongly Agree

Organization staff get recognition for implementing smoke-free environment promotion to reduce tobacco use. [B1]

◯ Strongly Disagree ◯ Disagree ◯ Neutral ◯ Agree ◯ Strongly Agree

The following are available to make promoting smoke-free environments work in our partnership:

	Strongly Disagree	Disagree	Neutral	Agree	Strongly Agree
equipment and materials [B2b]	◯	◯	◯	◯	◯
public awareness/need [B2b]	◯	◯	◯	◯	◯
staff buy-in or support [B2b]	◯	◯	◯	◯	◯
sufficient staffing [B2b]	◯	◯	◯	◯	◯
data systems/ IT support [B2b]	◯	◯	◯	◯	◯

Promoting smoke-free environments is more effective than our prior practices for reducing tobacco use. [A1]

◯ Strongly Disagree ◯ Disagree ◯ Neutral ◯ Agree ◯ Strongly Agree

It is difficult to train staff to implement smoke-free environment promotion. [A2]

◯ Strongly Disagree ◯ Disagree ◯ Neutral ◯ Agree ◯ Strongly Agree

Overall, I believe that it was complicated to implement smoke-free environment promotion. [A2]

◯ Strongly Disagree ◯ Disagree ◯ Neutral ◯ Agree ◯ Strongly Agree

I believe that promoting smoke-free environments has required my organization to make substantial changes to our previous practice. [A2]

◯ Strongly Disagree ◯ Disagree ◯ Neutral ◯ Agree ◯ Strongly Agree

Promoting smoke-free environments has required more work than can be done with current funding. [A2]

◯ Strongly Disagree ◯ Disagree ◯ Neutral ◯ Agree ◯ Strongly Agree

Using tobacco cessation couseling to reduce tobacco use is a top priority of the organization. [B1b]

◯ Strongly Disagree ◯ Disagree ◯ Neutral ◯ Agree ◯ Strongly Agree

Using tobacco cessation counseling to reduce tobacco use is compatible with current activities in the organization. [B1a]

◯ Strongly Disagree ◯ Disagree ◯ Neutral ◯ Agree ◯ Strongly Agree

I think that using tobacco cessation counseling to reduce tobacco use fits well with the way I like to work. [B1a]

◯ Strongly Disagree ◯ Disagree ◯ Neutral ◯ Agree ◯ Strongly Agree

Organization leaders establish clear goals for using tobacco cessation counseling to reduce tobacco use. [B1c]

◯ Strongly Disagree ◯ Disagree ◯ Neutral ◯ Agree ◯ Strongly Agree

Organization leaders hold staff members accountable for achieving results of tobacco cessation counseling. [B1c]

◯ Strongly Disagree ◯ Disagree ◯ Neutral ◯ Agree ◯ Strongly Agree

Organization staff are expected to help meet goals related to tobacco cessation counseling. [B1c]

◯ Strongly Disagree ◯ Disagree ◯ Neutral ◯ Agree ◯ Strongly Agree

Organization staff get the support they need to implement tobacco cessation counseling. [B1]

◯ Strongly Disagree ◯ Disagree ◯ Neutral ◯ Agree ◯ Strongly Agree

Organization staff get recognition for implementing tobacco cessation counseling to reduce tobacco use. [B1]

◯ Strongly Disagree ◯ Disagree ◯ Neutral ◯ Agree ◯ Strongly Agree

The following are available to make tobacco cessation counseling work in our partnership:

	Strongly Disagree	Disagree	Neutral	Agree	Strongly Agree
equipment and materials [B2b]	◯	◯	◯	◯	◯
public awareness/need [B2b]	◯	◯	◯	◯	◯
staff buy-in or support [B2b]	◯	◯	◯	◯	◯
sufficient staffing [B2b]	◯	◯	◯	◯	◯
data systems/IT support [B2b]	◯	◯	◯	◯	◯

Using tobacco cessation counseling is more effective than our prior practices for reducing tobacco use. [A1]

◯ Strongly Disagree ◯ Disagree ◯ Neutral ◯ Agree ◯ Strongly Agree

It is difficult to train staff to implement tobacco cessation counseling. [A2]

◯ Strongly Disagree ◯ Disagree ◯ Neutral ◯ Agree ◯ Strongly Agree

Overall, I believe that it was complicated to implement tobacco cessation counseling. [A2]

◯ Strongly Disagree ◯ Disagree ◯ Neutral ◯ Agree ◯ Strongly Agree

I believe that using tobacco cessation counseling has required my organization to make substantial changes to our previous practice. [A2]

◯ Strongly Disagree ◯ Disagree ◯ Neutral ◯ Agree ◯ Strongly Agree

Tobacco cessation counseling has required more work than can be done with current funding. [A2]

◯ Strongly Disagree ◯ Disagree ◯ Neutral ◯ Agree ◯ Strongly Agree

Hypertension

Did your partnership set goals around hypertension through the PWTF?

- ◯ Yes
- ◯ No

We are interested in YOUR involvement with the following best practices interventions for PWTF. Involvement could include coordinating & supporting implementation, making referrals, delivering interventions, conducting outreach, providing technical assistance, or supplying a venue/space.

Is your partnership implementing evidence-based guidelines for hypertension screening?

- ◯ Our partnership is working on this intervention, but I am not involved in implementation.
- ◯ No, our partnership is not implementing this intervention.
- ◯ Yes, we are in the early stage of implementation.
- ◯ Yes, we have implemented this strategy, but inconsistently.
- ◯ Yes, we have implemented this intervention fully and systematically.
- ◯ I don't know.

Is your partnership implementing chronic disease self-management programs to address hypertension?

- ◯ Our partnership is working on this intervention, but I am not involved in implementation.
- ◯ No, our partnership is not implementing this intervention.
- ◯ Yes, we are in the early stage of implementation.
- ◯ Yes, we have implemented this strategy, but inconsistently.
- ◯ Yes, we have implemented this intervention fully and systematically.
- ◯ I don't know.

Is your partnership implementing YMCA's Diabetes Prevention Program (YDPP) or National Diabetes Prevention Program (NDPP) for patients with hypertension?

- ◯ Our partnership is working on this intervention, but I am not involved in implementation.
- ◯ No, our partnership is not implementing this intervention.
- ◯ Yes, we are in the early stage of implementation.
- ◯ Yes, we have implemented this strategy, but inconsistently.
- ◯ Yes, we have implemented this intervention fully and systematically.
- ◯ I don't know.

Is your partnership implementing self-measured blood pressure monitoring with additional support to address hypertension?

- ◯ Our partnership is working on this intervention, but I am not involved in implementation.
- ◯ No, our partnership is not implementing this intervention.
- ◯ Yes, we are in the early stage of implementation.
- ◯ Yes, we have implemented this strategy, but inconsistently.
- ◯ Yes, we have implemented this intervention fully and systematically.
- ◯ I don't know.

Please select the answer that describes how much you agree or disagree with the following statements. We are interested in your experience implementing hypertension strategies, so there are no right or wrong answers to these questions.

Using evidence-based screening guidelines to reduce hypertension is a top priority of the organization.

◯ Strongly Disagree ◯ Disagree ◯ Neutral ◯ Agree ◯ Strongly Agree

Using evidence-based screening guidelines to reduce hypertension is compatible with current activities in the organization.

◯ Strongly Disagree ◯ Disagree ◯ Neutral ◯ Agree ◯ Strongly Agree

I think that using evidence-based screening guidelines to reduce hypertension fits well with the way I like to work.

◯ Strongly Disagree ◯ Disagree ◯ Neutral ◯ Agree ◯ Strongly Agree

Organization leaders establish clear goals for using evidence-based screening guidelines to reduce hypertension.

◯ Strongly Disagree ◯ Disagree ◯ Neutral ◯ Agree ◯ Strongly Agree

Organization leaders hold staff members accountable for achieving results of evidence-based guidelines for hypertension screening.

◯ Strongly Disagree ◯ Disagree ◯ Neutral ◯ Agree ◯ Strongly Agree

Organization staff are expected to help meet goals related to evidence-based guidelines for hypertension screening.

◯ Strongly Disagree ◯ Disagree ◯ Neutral ◯ Agree ◯ Strongly Agree

Organization staff get the support they need to implement evidence-based guidelines for hypertension screening.

◯ Strongly Disagree ◯ Disagree ◯ Neutral ◯ Agree ◯ Strongly Agree

Organization staff get recognition for implementing evidence-based screening guidelines to reduce hypertension.

◯ Strongly Disagree ◯ Disagree ◯ Neutral ◯ Agree ◯ Strongly Agree

The following are available to make evidence-based guidelines for hypertension screening work in our partnership:

	Strongly Disagree	Disagree	Neutral	Agree	Strongly Agree
equipment and materials	◯	◯	◯	◯	◯
public awareness/need	◯	◯	◯	◯	◯
staff buy-in or support	◯	◯	◯	◯	◯
sufficient staffing	◯	◯	◯	◯	◯
data systems/IT support	◯	◯	◯	◯	◯

Using evidence-based screening guidelines is more effective than our prior practices for reducing hypertension.

◯ Strongly Disagree ◯ Disagree ◯ Neutral ◯ Agree ◯ Strongly Agree

It is difficult to train staff to implement evidence-based guidelines for hypertension screening.

◯ Strongly Disagree ◯ Disagree ◯ Neutral ◯ Agree ◯ Strongly Agree

Overall, I believe that it was complicated to implement evidence-based guidelines for hypertension screening.

◯ Strongly Disagree ◯ Disagree ◯ Neutral ◯ Agree ◯ Strongly Agree

I believe that using evidence-based guidelines for hypertension screening has required my organization to make substantial changes to our previous practice.

◯ Strongly Disagree ◯ Disagree ◯ Neutral ◯ Agree ◯ Strongly Agree

Evidence-based guidelines for hypertension screening have required more work than can be done with current funding.

◯ Strongly Disagree ◯ Disagree ◯ Neutral ◯ Agree ◯ Strongly Agree

Using chronic disease self-management programs to reduce hypertension is a top priority of the organization.

◯ Strongly Disagree ◯ Disagree ◯ Neutral ◯ Agree ◯ Strongly Agree

Using chronic disease self-management programs to reduce hypertension is compatible with current activities in the organization.

◯ Strongly Disagree ◯ Disagree ◯ Neutral ◯ Agree ◯ Strongly Agree

I think that using chronic disease self-management programs to reduce hypertension fits well with the way I like to work.

◯ Strongly Disagree ◯ Disagree ◯ Neutral ◯ Agree ◯ Strongly Agree

Organization leaders establish clear goals for using chronic disease self-management programs to reduce hypertension.

◯ Strongly Disagree ◯ Disagree ◯ Neutral ◯ Agree ◯ Strongly Agree

Organization leaders hold staff members accountable for achieving results of chronic disease self-management programs.

◯ Strongly Disagree ◯ Disagree ◯ Neutral ◯ Agree ◯ Strongly Agree

Organization staff are expected to help meet goals related to chronic disease self-management programs.

◯ Strongly Disagree ◯ Disagree ◯ Neutral ◯ Agree ◯ Strongly Agree

Organization staff get the support they need to implement chronic disease self-management programs.

◯ Strongly Disagree ◯ Disagree ◯ Neutral ◯ Agree ◯ Strongly Agree

Organization staff get recognition for implementing chronic disease self-management programs to reduce hypertension.

◯ Strongly Disagree ◯ Disagree ◯ Neutral ◯ Agree ◯ Strongly Agree

The following are available to make chronic disease self-management programs work in our partnership:

	Strongly Disagree	Disagree	Neutral	Agree	Strongly Agree
equipment and materials	◯	◯	◯	◯	◯
public awareness/need	◯	◯	◯	◯	◯
staff buy-in or support	◯	◯	◯	◯	◯
sufficient staffing	◯	◯	◯	◯	◯
data systems/IT support	◯	◯	◯	◯	◯

Using chronic disease self-management programs is more effective than our prior practices for reducing hypertension.

◯ Strongly Disagree ◯ Disagree ◯ Neutral ◯ Agree ◯ Strongly Agree

It is difficult to train staff to implement chronic disease self-management programs.

◯ Strongly Disagree ◯ Disagree ◯ Neutral ◯ Agree ◯ Strongly Agree

Overall, I believe that it was complicated to implement chronic disease self-management programs.

◯ Strongly Disagree ◯ Disagree ◯ Neutral ◯ Agree ◯ Strongly Agree

I believe that using chronic disease self-management programs has required my organization to make substantial changes to our previous practice.

◯ Strongly Disagree ◯ Disagree ◯ Neutral ◯ Agree ◯ Strongly Agree

Chronic disease self-management programs have required more work than can be done with current funding.

◯ Strongly Disagree ◯ Disagree ◯ Neutral ◯ Agree ◯ Strongly Agree

Using YMCA's Diabetes Prevention Program or National Diabetes Prevention Program to reduce hypertension is a top priority of the organization.

○ Strongly Disagree ○ Disagree ○ Neutral ○ Agree ○ Strongly Agree

Using YMCA's Diabetes Prevention Program or National Diabetes Prevention Program to reduce hypertension is compatible with current activities in the organization.

○ Strongly Disagree ○ Disagree ○ Neutral ○ Agree ○ Strongly Agree

I think that using YMCA's Diabetes Prevention Program or National Diabetes Prevention Program to reduce hypertension fits well with the way I like to work.

○ Strongly Disagree ○ Disagree ○ Neutral ○ Agree ○ Strongly Agree

Organization leaders establish clear goals for using YMCA's Diabetes Prevention Program or National Diabetes Prevention Program to reduce hypertension.

○ Strongly Disagree ○ Disagree ○ Neutral ○ Agree ○ Strongly Agree

Organization leaders hold staff members accountable for achieving results of YMCA's Diabetes Prevention Program or National Diabetes Prevention Program.

○ Strongly Disagree ○ Disagree ○ Neutral ○ Agree ○ Strongly Agree

Organization staff are expected to help meet goals related to YMCA's Diabetes Prevention Program or National Diabetes Prevention Program.

○ Strongly Disagree ○ Disagree ○ Neutral ○ Agree ○ Strongly Agree

Organization staff get the support they need to implement YMCA's Diabetes Prevention Program or National Diabetes Prevention Program.

○ Strongly Disagree ○ Disagree ○ Neutral ○ Agree ○ Strongly Agree

Organization staff get recognition for implementing YMCA's Diabetes Prevention Program or National Diabetes Prevention Program to reduce hypertension.

○ Strongly Disagree ○ Disagree ○ Neutral ○ Agree ○ Strongly Agree

The following are available to make YMCA's Diabetes Prevention Program or National Diabetes Prevention Program work in our partnership

	Strongly Disagree	Disagree	Neutral	Agree	Strongly Agree
equipment and materials	○	○	○	○	○
public awareness/need	○	○	○	○	○
staff buy-in or support	○	○	○	○	○
sufficient staffing	○	○	○	○	○
data systems/IT support	○	○	○	○	○

Using YMCA's Diabetes Prevention Program or National Diabetes Prevention Program is more effective than our prior practices for reducing hypertension.

○ Strongly Disagree ○ Disagree ○ Neutral ○ Agree ○ Strongly Agree

It is difficult to train staff to implement YMCA's Diabetes Prevention Program or National Diabetes Prevention Program.

○ Strongly Disagree ○ Disagree ○ Neutral ○ Agree ○ Strongly Agree

Overall, I believe that it was complicated to implement YMCA's Diabetes Prevention Program or National Diabetes Prevention Program.

○ Strongly Disagree ○ Disagree ○ Neutral ○ Agree ○ Strongly Agree

I believe that using YMCA's Diabetes Prevention Program or National Diabetes Prevention Program has required my organization to make substantial changes to our previous practice.

○ Strongly Disagree ○ Disagree ○ Neutral ○ Agree ○ Strongly Agree

YMCA's Diabetes Prevention Program or National Diabetes Prevention Program has required more work than can be done with current funding.

○ Strongly Disagree ○ Disagree ○ Neutral ○ Agree ○ Strongly Agree

Using self-measured blood pressure monitoring with additional support to reduce hypertension is a top priority of the organization.

◯ Strongly Disagree ◯ Disagree ◯ Neutral ◯ Agree ◯ Strongly Agree

Using self-measured blood pressure monitoring with additional support to reduce hypertension is compatible with current activities in the organization.

◯ Strongly Disagree ◯ Disagree ◯ Neutral ◯ Agree ◯ Strongly Agree

I think that using self-measured blood pressure monitoring with additional support to reduce hypertension fits well with the way I like to work.

◯ Strongly Disagree ◯ Disagree ◯ Neutral ◯ Agree ◯ Strongly Agree

Organization leaders establish clear goals for using self-measured blood pressure monitoring with additional support to reduce hypertension.

◯ Strongly Disagree ◯ Disagree ◯ Neutral ◯ Agree ◯ Strongly Agree

Organization leaders hold staff members accountable for achieving results of self-measured blood pressure monitoring with additional support.

◯ Strongly Disagree ◯ Disagree ◯ Neutral ◯ Agree ◯ Strongly Agree

Organization staff are expected to help meet goals related to self-measured blood pressure monitoring with additional support.

◯ Strongly Disagree ◯ Disagree ◯ Neutral ◯ Agree ◯ Strongly Agree

Organization staff get the support they need to implement self-measured blood pressure monitoring with additional support.

◯ Strongly Disagree ◯ Disagree ◯ Neutral ◯ Agree ◯ Strongly Agree

Organization staff get recognition for implementing self-measured blood pressure monitoring with additional support to reduce hypertension.

◯ Strongly Disagree ◯ Disagree ◯ Neutral ◯ Agree ◯ Strongly Agree

The following are available to make self-measured blood pressure monitoring with additional support work in our partnership:

	Strongly Disagree	Disagree	Neutral	Agree	Strongly Agree
equipment and materials	◯	◯	◯	◯	◯
public awareness/need	◯	◯	◯	◯	◯
staff buy-in or support	◯	◯	◯	◯	◯
sufficient staffing	◯	◯	◯	◯	◯
data systems/IT support	◯	◯	◯	◯	◯

Using self-measured blood pressure monitoring with additional support is more effective than our prior practices for reducing hypertension.

◯ Strongly Disagree ◯ Disagree ◯ Neutral ◯ Agree ◯ Strongly Agree

It is difficult to train staff to implement self-measured blood pressure monitoring with additional support.

◯ Strongly Disagree ◯ Disagree ◯ Neutral ◯ Agree ◯ Strongly Agree

Overall, I believe that it was complicated to implement self-measured blood pressure monitoring with additional support.

◯ Strongly Disagree ◯ Disagree ◯ Neutral ◯ Agree ◯ Strongly Agree

I believe that using self-measured blood pressure monitoring with additional support has required my organization to make substantial changes to our previous practice.

◯ Strongly Disagree ◯ Disagree ◯ Neutral ◯ Agree ◯ Strongly Agree

Self-measured blood pressure monitoring with additional support has required more work than can be done with current funding.

◯ Strongly Disagree ◯ Disagree ◯ Neutral ◯ Agree ◯ Strongly Agree

Pediatric Asthma

Did your partnership set goals around pediatric asthma through the PWTF?

- ◯ Yes
- ◯ No

We are interested in YOUR involvement with the following best practices interventions for PWTF. Involvement could include coordinating & supporting implementation, making referrals, delivering interventions, conducting outreach, providing technical assistance, or supplying a venue/space.

Is your partnership implementing care management for high-risk asthma patients to address pediatric asthma?

- ◯ Our partnership is working on this intervention, but I am not involved in implementation.
- ◯ No, our partnership is not implementing this intervention.
- ◯ Yes, we are in the early stage of implementation.
- ◯ Yes, we have implemented this strategy, but inconsistently.
- ◯ Yes, we have implemented this intervention fully and systematically.
- ◯ I don't know.

Is your partnership implementing asthma self-management in primary care to address pediatric asthma?

- ◯ Our partnership is working on this intervention, but I am not involved in implementation.
- ◯ No, our partnership is not implementing this intervention.
- ◯ Yes, we are in the early stage of implementation.
- ◯ Yes, we have implemented this strategy, but inconsistently.
- ◯ Yes, we have implemented this intervention fully and systematically.
- ◯ I don't know.

Is your partnership implementing a home-based multi-trigger, multi-component intervention to address pediatric asthma?

- ◯ Our partnership is working on this intervention, but I am not involved in implementation.
- ◯ No, our partnership is not implementing this intervention.
- ◯ Yes, we are in the early stage of implementation.
- ◯ Yes, we have implemented this strategy, but inconsistently.
- ◯ Yes, we have implemented this intervention fully and systematically.
- ◯ I don't know.

Is your partnership implementing comprehensive head start-based asthma programs to address pediatric asthma?

- ◯ Our partnership is working on this intervention, but I am not involved in implementation.
- ◯ No, our partnership is not implementing this intervention.
- ◯ Yes, we are in the early stage of implementation.
- ◯ Yes, we have implemented this strategy, but inconsistently.
- ◯ Yes, we have implemented this intervention fully and systematically.
- ◯ I don't know.

Is your partnership implementing comprehensive school-based asthma programs to address pediatric asthma?

◯ Our partnership is working on this intervention, but I am not involved in implementation.
◯ No, our partnership is not implementing this intervention.
◯ Yes, we are in the early stage of implementation.
◯ Yes, we have implemented this strategy, but inconsistently.
◯ Yes, we have implemented this intervention fully and systematically.
◯ I don't know.

Please select the answer that describes how much you agree or disagree with the following statements. We are interested in your experience implementing pediatric asthma strategies, so there are no right or wrong answers to these questions.

Using care management for high-risk asthma patients to reduce pediatric asthma is a top priority of the organization.

◯ Strongly Disagree ◯ Disagree ◯ Neutral ◯ Agree ◯ Strongly Agree

Using care management for high-risk asthma patients to reduce pediatric asthma is compatible with current activities in the organization.

◯ Strongly Disagree ◯ Disagree ◯ Neutral ◯ Agree ◯ Strongly Agree

I think that using care management for high-risk asthma patients to reduce pediatric asthma fits well with the way I like to work.

◯ Strongly Disagree ◯ Disagree ◯ Neutral ◯ Agree ◯ Strongly Agree

Organization leaders establish clear goals for using care management for high-risk asthma patients to reduce pediatric asthma.

◯ Strongly Disagree ◯ Disagree ◯ Neutral ◯ Agree ◯ Strongly Agree

Organization leaders hold staff members accountable for achieving results of care management for high-risk asthma patients.

◯ Strongly Disagree ◯ Disagree ◯ Neutral ◯ Agree ◯ Strongly Agree

Organization staff are expected to help meet goals related to care management for high-risk asthma patients.

◯ Strongly Disagree ◯ Disagree ◯ Neutral ◯ Agree ◯ Strongly Agree

Organization staff get the support they need to implement care management for high-risk asthma patients.

◯ Strongly Disagree ◯ Disagree ◯ Neutral ◯ Agree ◯ Strongly Agree

Organization staff get recognition for implementing care management for high-risk asthma patients to reduce pediatric asthma.

◯ Strongly Disagree ◯ Disagree ◯ Neutral ◯ Agree ◯ Strongly Agree

The following are available to make care management for high-risk asthma patients work in our partnership:

	Strongly Disagree	Disagree	Neutral	Agree	Strongly Agree
equipment and materials	◯	◯	◯	◯	◯
public awareness/need	◯	◯	◯	◯	◯
staff buy-in or support	◯	◯	◯	◯	◯
sufficient staffing	◯	◯	◯	◯	◯
data systems/IT support	◯	◯	◯	◯	◯

Using care management for high-risk asthma patients is more effective than our prior practices for reducing pediatric asthma.

◯ Strongly Disagree ◯ Disagree ◯ Neutral ◯ Agree ◯ Strongly Agree

It is difficult to train staff to implement care management for high-risk asthma patients.

◯ Strongly Disagree ◯ Disagree ◯ Neutral ◯ Agree ◯ Strongly Agree

Overall, I believe that it was complicated to implement care management for high-risk asthma patients.

◯ Strongly Disagree ◯ Disagree ◯ Neutral ◯ Agree ◯ Strongly Agree

I believe that using care management for high-risk asthma patients has required my organization to make substantial changes to our previous practice.

◯ Strongly Disagree ◯ Disagree ◯ Neutral ◯ Agree ◯ Strongly Agree

Care management for high-risk asthma patients has required more work than can be done with current funding.

◯ Strongly Disagree ◯ Disagree ◯ Neutral ◯ Agree ◯ Strongly Agree

Using asthma self-management in primary care to reduce pediatric asthma is a top priority of the organization.

◯ Strongly Disagree ◯ Disagree ◯ Neutral ◯ Agree ◯ Strongly Agree

Using asthma self-management in primary care to reduce pediatric asthma is compatible with current activities in the organization.

◯ Strongly Disagree ◯ Disagree ◯ Neutral ◯ Agree ◯ Strongly Agree

I think that using asthma self-management in primary care to reduce pediatric asthma fits well with the way I like to work.

◯ Strongly Disagree ◯ Disagree ◯ Neutral ◯ Agree ◯ Strongly Agree

Organization leaders establish clear goals for using asthma self-management in primary care to reduce pediatric asthma.

◯ Strongly Disagree ◯ Disagree ◯ Neutral ◯ Agree ◯ Strongly Agree

Organization leaders hold staff members accountable for achieving results of asthma self-management in primary care.

◯ Strongly Disagree ◯ Disagree ◯ Neutral ◯ Agree ◯ Strongly Agree

Organization staff are expected to help meet goals related to asthma self-management in primary care.

◯ Strongly Disagree ◯ Disagree ◯ Neutral ◯ Agree ◯ Strongly Agree

Organization staff get the support they need to implement asthma self-management in primary care.

◯ Strongly Disagree ◯ Disagree ◯ Neutral ◯ Agree ◯ Strongly Agree

Organization staff get recognition for implementing asthma self-management in primary care to reduce pediatric asthma.

◯ Strongly Disagree ◯ Disagree ◯ Neutral ◯ Agree ◯ Strongly Agree

The following are available to make asthma self-management in primary care work in our partnership:

	Strongly Disagree	Disagree	Neutral	Agree	Strongly Agree
equipment and materials	◯	◯	◯	◯	◯
public awareness/need	◯	◯	◯	◯	◯
staff buy-in or support	◯	◯	◯	◯	◯
sufficient staffing	◯	◯	◯	◯	◯
data systems/IT support	◯	◯	◯	◯	◯

Using asthma self-management in primary care is more effective than our prior practices for reducing pediatric asthma.

◯ Strongly Disagree ◯ Disagree ◯ Neutral ◯ Agree ◯ Strongly Agree

It is difficult to train staff to implement asthma self-management in primary care.

◯ Strongly Disagree ◯ Disagree ◯ Neutral ◯ Agree ◯ Strongly Agree

Overall, I believe that it was complicated to implement asthma self-management in primary care.

◯ Strongly Disagree ◯ Disagree ◯ Neutral ◯ Agree ◯ Strongly Agree

I believe that using asthma self-management in primary care has required my organization to make substantial changes to our previous practice.

◯ Strongly Disagree ◯ Disagree ◯ Neutral ◯ Agree ◯ Strongly Agree

Asthma self-management in primary care has required more work than can be done with current funding.

◯ Strongly Disagree ◯ Disagree ◯ Neutral ◯ Agree ◯ Strongly Agree

Using a home-based multi-trigger, multi-component intervention to reduce pediatric asthma is a top priority of the organization.

◯ Strongly Disagree ◯ Disagree ◯ Neutral ◯ Agree ◯ Strongly Agree

Using a home-based multi-trigger, multi-component intervention to reduce pediatric asthma is compatible with current activities in the organization.

◯ Strongly Disagree ◯ Disagree ◯ Neutral ◯ Agree ◯ Strongly Agree

I think that using a home-based multi-trigger, multi-component intervention to reduce pediatric asthma fits well with the way I like to work.

◯ Strongly Disagree ◯ Disagree ◯ Neutral ◯ Agree ◯ Strongly Agree

Organization leaders establish clear goals for using a home-based multi-trigger, multi-component intervention to reduce pediatric asthma.

◯ Strongly Disagree ◯ Disagree ◯ Neutral ◯ Agree ◯ Strongly Agree

Organization leaders hold staff members accountable for achieving results of a home-based multi-trigger, multi-component intervention.

◯ Strongly Disagree ◯ Disagree ◯ Neutral ◯ Agree ◯ Strongly Agree

Organization staff are expected to help meet goals related to a home-based multi-trigger, multi-component intervention.

◯ Strongly Disagree ◯ Disagree ◯ Neutral ◯ Agree ◯ Strongly Agree

Organization staff get the support they need to implement a home-based multi-trigger, multi-component intervention.

◯ Strongly Disagree ◯ Disagree ◯ Neutral ◯ Agree ◯ Strongly Agree

Organization staff get recognition for implementing a home-based multi-trigger, multi-component intervention to reduce pediatric asthma.

◯ Strongly Disagree ◯ Disagree ◯ Neutral ◯ Agree ◯ Strongly Agree

The following are available to make a home-based multi-trigger, multi-component intervention work in our partnership:

	Strongly Disagree	Disagree	Neutral	Agree	Strongly Agree
equipment and materials	◯	◯	◯	◯	◯
public awareness/need	◯	◯	◯	◯	◯
staff buy-in or support	◯	◯	◯	◯	◯
sufficient staffing	◯	◯	◯	◯	◯
data systems/IT support	◯	◯	◯	◯	◯

Using a home-based multi-trigger, multi-component intervention is more effective than our prior practices for reducing pediatric asthma.

◯ Strongly Disagree ◯ Disagree ◯ Neutral ◯ Agree ◯ Strongly Agree

It is difficult to train staff to implement a home-based multi-trigger, multi-component intervention.

◯ Strongly Disagree ◯ Disagree ◯ Neutral ◯ Agree ◯ Strongly Agree

Overall, I believe that it was complicated to implement a home-based multi-trigger, multi-component intervention.

◯ Strongly Disagree ◯ Disagree ◯ Neutral ◯ Agree ◯ Strongly Agree

I believe that using a home-based multi-trigger, multi-component intervention has required my organization to make substantial changes to our previous practice.

◯ Strongly Disagree ◯ Disagree ◯ Neutral ◯ Agree ◯ Strongly Agree

A home-based multi-trigger, multi-component intervention has required more work than can be done with current funding.

◯ Strongly Disagree ◯ Disagree ◯ Neutral ◯ Agree ◯ Strongly Agree

Using comprehensive head start-based asthma programs to reduce pediatric asthma is a top priority of the organization.

◯ Strongly Disagree ◯ Disagree ◯ Neutral ◯ Agree ◯ Strongly Agree

Using comprehensive head start-based asthma programs to reduce pediatric asthma is compatible with current activities in the organization.

◯ Strongly Disagree ◯ Disagree ◯ Neutral ◯ Agree ◯ Strongly Agree

I think that using comprehensive head start-based asthma programs to reduce pediatric asthma fits well with the way I like to work.

◯ Strongly Disagree ◯ Disagree ◯ Neutral ◯ Agree ◯ Strongly Agree

Organization leaders establish clear goals for using comprehensive head start-based asthma programs to reduce pediatric asthma.

◯ Strongly Disagree ◯ Disagree ◯ Neutral ◯ Agree ◯ Strongly Agree

Organization leaders hold staff members accountable for achieving results of comprehensive head start-based asthma programs.

◯ Strongly Disagree ◯ Disagree ◯ Neutral ◯ Agree ◯ Strongly Agree

Organization staff are expected to help meet goals related to comprehensive head start-based asthma programs.

◯ Strongly Disagree ◯ Disagree ◯ Neutral ◯ Agree ◯ Strongly Agree

Organization staff get the support they need to implement comprehensive head start-based asthma programs.

◯ Strongly Disagree ◯ Disagree ◯ Neutral ◯ Agree ◯ Strongly Agree

Organization staff get recognition for implementing comprehensive head start-based asthma programs to reduce pediatric asthma.

◯ Strongly Disagree ◯ Disagree ◯ Neutral ◯ Agree ◯ Strongly Agree

The following are available to make comprehensive head start-based asthma programs work in our partnership:

	Strongly Disagree	Disagree	Neutral	Agree	Strongly Agree
equipment and materials	◯	◯	◯	◯	◯
public awareness/need	◯	◯	◯	◯	◯
staff buy-in or support	◯	◯	◯	◯	◯
sufficient staffing	◯	◯	◯	◯	◯
data systems/IT support	◯	◯	◯	◯	◯

Using comprehensive head start-based asthma programs is more effective than our prior practices for reducing pediatric asthma.

◯ Strongly Disagree ◯ Disagree ◯ Neutral ◯ Agree ◯ Strongly Agree

It is difficult to train staff to implement comprehensive head start-based asthma programs.

◯ Strongly Disagree ◯ Disagree ◯ Neutral ◯ Agree ◯ Strongly Agree

Overall, I believe that it was complicated to implement comprehensive head start-based asthma programs.

◯ Strongly Disagree ◯ Disagree ◯ Neutral ◯ Agree ◯ Strongly Agree

I believe that using comprehensive head start-based asthma programs has required my organization to make substantial changes to our previous practice.

◯ Strongly Disagree ◯ Disagree ◯ Neutral ◯ Agree ◯ Strongly Agree

Comprehensive head start-based asthma programs have required more work than can be done with current funding.

◯ Strongly Disagree ◯ Disagree ◯ Neutral ◯ Agree ◯ Strongly Agree

Using comprehensive school-based asthma programs to reduce pediatric asthma is a top priority of the organization.

○ Strongly Disagree ○ Disagree ○ Neutral ○ Agree ○ Strongly Agree

Using comprehensive school-based asthma programs to reduce pediatric asthma is compatible with current activities in the organization.

○ Strongly Disagree ○ Disagree ○ Neutral ○ Agree ○ Strongly Agree

I think that using comprehensive school-based asthma programs to reduce pediatric asthma fits well with the way I like to work.

○ Strongly Disagree ○ Disagree ○ Neutral ○ Agree ○ Strongly Agree

Organization leaders establish clear goals for using comprehensive school-based asthma programs to reduce pediatric asthma.

○ Strongly Disagree ○ Disagree ○ Neutral ○ Agree ○ Strongly Agree

Organization leaders hold staff members accountable for achieving results of comprehensive school-based asthma programs.

○ Strongly Disagree ○ Disagree ○ Neutral ○ Agree ○ Strongly Agree

Organization staff are expected to help meet goals related to comprehensive school-based asthma programs.

○ Strongly Disagree ○ Disagree ○ Neutral ○ Agree ○ Strongly Agree

Organization staff get the support they need to implement comprehensive school-based asthma programs.

○ Strongly Disagree ○ Disagree ○ Neutral ○ Agree ○ Strongly Agree

Organization staff get recognition for implementing comprehensive school-based asthma programs to reduce pediatric asthma.

○ Strongly Disagree ○ Disagree ○ Neutral ○ Agree ○ Strongly Agree

The following are available to make comprehensive school-based asthma programs work in our partnership:

	Strongly Disagree	Disagree	Neutral	Agree	Strongly Agree
equipment and materials	◯	◯	◯	◯	◯
public awareness/need	◯	◯	◯	◯	◯
staff buy-in or support	◯	◯	◯	◯	◯
sufficient staffing	◯	◯	◯	◯	◯
data systems/IT support	◯	◯	◯	◯	◯

Using comprehensive school-based asthma programs is more effective than our prior practices for reducing pediatric asthma.

◯ Strongly Disagree ◯ Disagree ◯ Neutral ◯ Agree ◯ Strongly Agree

It is difficult to train staff to implement comprehensive school-based asthma programs.

◯ Strongly Disagree ◯ Disagree ◯ Neutral ◯ Agree ◯ Strongly Agree

Overall, I believe that it was complicated to implement comprehensive school-based asthma programs.

◯ Strongly Disagree ◯ Disagree ◯ Neutral ◯ Agree ◯ Strongly Agree

I believe that using comprehensive school-based asthma programs has required my organization to make substantial changes to our previous practice.

◯ Strongly Disagree ◯ Disagree ◯ Neutral ◯ Agree ◯ Strongly Agree

Comprehensive school-based asthma programs have required more work than can be done with current funding.

◯ Strongly Disagree ◯ Disagree ◯ Neutral ◯ Agree ◯ Strongly Agree

Falls in Older Adults

Did your partnership set goals around falls in older adults through the PWTF?

- ◯ Yes
- ◯ No

We are interested in YOUR involvement with the following best practices interventions for PWTF. Involvement could include coordinating & supporting implementation, making referrals, delivering interventions, conducting outreach, providing technical assistance, or supplying a venue/space.

Is your partnership implementing Stopping Elderly Accidents, Deaths, and Injuries (STEADI) clinical risk assessment to address falls in older adults?

- ◯ Our partnership is working on this intervention, but I am not involved in implementation.
- ◯ No, our partnership is not implementing this intervention.
- ◯ Yes, we are in the early stage of implementation.
- ◯ Yes, we have implemented this strategy, but inconsistently.
- ◯ Yes, we have implemented this intervention fully and systematically.
- ◯ I don't know.

Is your partnership implementing Tai Chi: Moving for Better Balance to address falls in older adults?

- ◯ Our partnership is working on this intervention, but I am not involved in implementation.
- ◯ No, our partnership is not implementing this intervention.
- ◯ Yes, we are in the early stage of implementation.
- ◯ Yes, we have implemented this strategy, but inconsistently.
- ◯ Yes, we have implemented this intervention fully and systematically.
- ◯ I don't know.

Is your partnership implementing A Matter of Balance to address falls in older adults?

- ◯ Our partnership is working on this intervention, but I am not involved in implementation.
- ◯ No, our partnership is not implementing this intervention.
- ◯ Yes, we are in the early stage of implementation.
- ◯ Yes, we have implemented this strategy, but inconsistently.
- ◯ Yes, we have implemented this intervention fully and systematically.
- ◯ I don't know.

Is your partnership implementing home safety assessments and modifications to address falls in older adults?

- ◯ Our partnership is working on this intervention, but I am not involved in implementation.
- ◯ No, our partnership is not implementing this intervention.
- ◯ Yes, we are in the early stage of implementation.
- ◯ Yes, we have implemented this strategy, but inconsistently.
- ◯ Yes, we have implemented this intervention fully and systematically.
- ◯ I don't know.

Please select the answer that describes how much you agree or disagree with the following statements. We are interested in your experience implementing falls in older adults strategies, so there are no right or wrong answers to these questions.

Using STEADI clinical risk assessment to reduce falls in older adults is a top priority of the organization.

◯ Strongly Disagree ◯ Disagree ◯ Neutral ◯ Agree ◯ Strongly Agree

Using STEADI clinical risk assessment to reduce falls in older adults is compatible with current activities in the organization.

◯ Strongly Disagree ◯ Disagree ◯ Neutral ◯ Agree ◯ Strongly Agree

I think that using STEADI clinical risk assessment to reduce falls in older adults fits well with the way I like to work.

◯ Strongly Disagree ◯ Disagree ◯ Neutral ◯ Agree ◯ Strongly Agree

Organization leaders establish clear goals for using STEADI clinical risk assessment to reduce falls in older adults.

◯ Strongly Disagree ◯ Disagree ◯ Neutral ◯ Agree ◯ Strongly Agree

Organization leaders hold staff members accountable for achieving results of STEADI clinical risk assessment.

◯ Strongly Disagree ◯ Disagree ◯ Neutral ◯ Agree ◯ Strongly Agree

Organization staff are expected to help meet goals related to STEADI clinical risk assessment.

◯ Strongly Disagree ◯ Disagree ◯ Neutral ◯ Agree ◯ Strongly Agree

Organization staff get the support they need to implement STEADI clinical risk assessment.

◯ Strongly Disagree ◯ Disagree ◯ Neutral ◯ Agree ◯ Strongly Agree

Organization staff get recognition for implementing STEADI clinical risk assessment to reduce falls in older adults.

◯ Strongly Disagree ◯ Disagree ◯ Neutral ◯ Agree ◯ Strongly Agree

The following are available to make STEADI clinical risk assessment work in our partnership:

	Strongly Disagree	Disagree	Neutral	Agree	Strongly Agree
equipment and materials	◯	◯	◯	◯	◯
public awareness/need	◯	◯	◯	◯	◯
staff buy-in or support	◯	◯	◯	◯	◯
sufficient staffing	◯	◯	◯	◯	◯
data systems/IT support	◯	◯	◯	◯	◯

Using STEADI clinical risk assessment is more effective than our prior practices for reducing falls in older adults.

◯ Strongly Disagree ◯ Disagree ◯ Neutral ◯ Agree ◯ Strongly Agree

It is difficult to train staff to implement STEADI clinical risk assessment.

◯ Strongly Disagree ◯ Disagree ◯ Neutral ◯ Agree ◯ Strongly Agree

Overall, I believe that it was complicated to implement STEADI clinical risk assessment.

◯ Strongly Disagree ◯ Disagree ◯ Neutral ◯ Agree ◯ Strongly Agree

I believe that using STEADI clinical risk assessment has required my organization to make substantial changes to our previous practice.

◯ Strongly Disagree ◯ Disagree ◯ Neutral ◯ Agree ◯ Strongly Agree

STEADI clinical risk assessment has required more work than can be done with current funding.

◯ Strongly Disagree ◯ Disagree ◯ Neutral ◯ Agree ◯ Strongly Agree

Using Tai Chi: Moving for Better Balance to reduce falls in older adults is a top priority of the organization.

◯ Strongly Disagree ◯ Disagree ◯ Neutral ◯ Agree ◯ Strongly Agree

Using Tai Chi: Moving for Better Balance to reduce falls in older adults is compatible with current activities in the organization.

◯ Strongly Disagree ◯ Disagree ◯ Neutral ◯ Agree ◯ Strongly Agree

I think that using Tai Chi: Moving for Better Balance to reduce falls in older adults fits well with the way I like to work.

◯ Strongly Disagree ◯ Disagree ◯ Neutral ◯ Agree ◯ Strongly Agree

Organization leaders establish clear goals for using Tai Chi: Moving for Better Balance to reduce falls in older adults.

◯ Strongly Disagree ◯ Disagree ◯ Neutral ◯ Agree ◯ Strongly Agree

Organization leaders hold staff members accountable for achieving results of Tai Chi: Moving for Better Balance.

◯ Strongly Disagree ◯ Disagree ◯ Neutral ◯ Agree ◯ Strongly Agree

Organization staff are expected to help meet goals related to Tai Chi: Moving for Better Balance.

◯ Strongly Disagree ◯ Disagree ◯ Neutral ◯ Agree ◯ Strongly Agree

Organization staff get the support they need to implement Tai Chi: Moving for Better Balance.

◯ Strongly Disagree ◯ Disagree ◯ Neutral ◯ Agree ◯ Strongly Agree

Organization staff get recognition for implementing Tai Chi: Moving for Better Balance to reduce falls in older adults.

◯ Strongly Disagree ◯ Disagree ◯ Neutral ◯ Agree ◯ Strongly Agree

The following are available to make Tai Chi: Moving for Better Balance work in our practice:

	Strongly Disagree	Disagree	Neutral	Agree	Strongly Agree
equipment and materials	◯	◯	◯	◯	◯
public awareness/need	◯	◯	◯	◯	◯
staff buy-in or support	◯	◯	◯	◯	◯
sufficient staffing	◯	◯	◯	◯	◯
data systems/IT support	◯	◯	◯	◯	◯

Using Tai Chi: Moving for Better Balance is more effective than our prior practices for reducing falls in older adults.

◯ Strongly Disagree ◯ Disagree ◯ Neutral ◯ Agree ◯ Strongly Agree

It is difficult to train staff to implement Tai Chi: Moving for Better Balance.

◯ Strongly Disagree ◯ Disagree ◯ Neutral ◯ Agree ◯ Strongly Agree

Overall, I believe that it was complicated to implement Tai Chi: Moving for Better Balance.

◯ Strongly Disagree ◯ Disagree ◯ Neutral ◯ Agree ◯ Strongly Agree

I believe that using Tai Chi: Moving for Better Balance has required my organization to make substantial changes to our previous practice.

◯ Strongly Disagree ◯ Disagree ◯ Neutral ◯ Agree ◯ Strongly Agree

Tai Chi: Moving for Better Balance has required more work than can be done with current funding.

◯ Strongly Disagree ◯ Disagree ◯ Neutral ◯ Agree ◯ Strongly Agree

Using A Matter of Balance to reduce falls in older adults is a top priority of the organization.

◯ Strongly Disagree ◯ Disagree ◯ Neutral ◯ Agree ◯ Strongly Agree

Using A Matter of Balance to reduce falls in older adults is compatible with current activities in the organization.

◯ Strongly Disagree ◯ Disagree ◯ Neutral ◯ Agree ◯ Strongly Agree

I think that using A Matter of Balance to reduce falls in older adults fits well with the way I like to work.

◯ Strongly Disagree ◯ Disagree ◯ Neutral ◯ Agree ◯ Strongly Agree

Organization leaders establish clear goals for using A Matter of Balance to reduce falls in older adults.

◯ Strongly Disagree ◯ Disagree ◯ Neutral ◯ Agree ◯ Strongly Agree

Organization leaders hold staff members accountable for achieving results of A Matter of Balance.

◯ Strongly Disagree ◯ Disagree ◯ Neutral ◯ Agree ◯ Strongly Agree

Organization staff are expected to help meet goals related to A Matter of Balance.

◯ Strongly Disagree ◯ Disagree ◯ Neutral ◯ Agree ◯ Strongly Agree

Organization staff get the support they need to implement A Matter of Balance.

◯ Strongly Disagree ◯ Disagree ◯ Neutral ◯ Agree ◯ Strongly Agree

Organization staff get recognition for implementing A Matter of Balance to reduce falls in older adults.

◯ Strongly Disagree ◯ Disagree ◯ Neutral ◯ Agree ◯ Strongly Agree

The following are available to make A Matter of Balance work in our partnership:

	Strongly Disagree	Disagree	Neutral	Agree	Strongly Agree
equipment and materials	◯	◯	◯	◯	◯
public awareness/need	◯	◯	◯	◯	◯
staff buy-in or support	◯	◯	◯	◯	◯
sufficient staffing	◯	◯	◯	◯	◯
data systems/IT support	◯	◯	◯	◯	◯

Using A Matter of Balance is more effective than our prior practices for reducing falls in older adults.

◯ Strongly Disagree ◯ Disagree ◯ Neutral ◯ Agree ◯ Strongly Agree

It is difficult to train staff to implement A Matter of Balance.

◯ Strongly Disagree ◯ Disagree ◯ Neutral ◯ Agree ◯ Strongly Agree

Overall, I believe that it was complicated to implement A Matter of Balance.

◯ Strongly Disagree ◯ Disagree ◯ Neutral ◯ Agree ◯ Strongly Agree

I believe that using A Matter of Balance has required my organization to make substantial changes to our previous practice.

◯ Strongly Disagree ◯ Disagree ◯ Neutral ◯ Agree ◯ Strongly Agree

A Matter of Balance has required more work than can be done with current funding.

◯ Strongly Disagree ◯ Disagree ◯ Neutral ◯ Agree ◯ Strongly Agree

Using home safety assessments and modification to reduce falls in older adults is a top priority of the organization.

◯ Strongly Disagree ◯ Disagree ◯ Neutral ◯ Agree ◯ Strongly Agree

Using home safety assessments and modification to reduce falls in older adults is compatible with current activities in the organization.

◯ Strongly Disagree ◯ Disagree ◯ Neutral ◯ Agree ◯ Strongly Agree

I think that using home safety assessments and modification to reduce falls in older adults fits well with the way I like to work.

◯ Strongly Disagree ◯ Disagree ◯ Neutral ◯ Agree ◯ Strongly Agree

Organization leaders establish clear goals for using home safety assessments and modification to reduce falls in older adults.

◯ Strongly Disagree ◯ Disagree ◯ Neutral ◯ Agree ◯ Strongly Agree

Organization leaders hold staff members accountable for achieving results of home safety assessments and modification.

◯ Strongly Disagree ◯ Disagree ◯ Neutral ◯ Agree ◯ Strongly Agree

Organization staff are expected to help meet goals related to home safety assessments and modification.

◯ Strongly Disagree ◯ Disagree ◯ Neutral ◯ Agree ◯ Strongly Agree

Organization staff get the support they need to implement home safety assessments and modification.

◯ Strongly Disagree ◯ Disagree ◯ Neutral ◯ Agree ◯ Strongly Agree

Organization staff get recognition for implementing home safety assessments and modification to reduce falls in older adults.

◯ Strongly Disagree ◯ Disagree ◯ Neutral ◯ Agree ◯ Strongly Agree

The following are available to make home safety assessments and modification work in our partnership:

	Strongly Disagree	Disagree	Neutral	Agree	Strongly Agree
equipment and materials	◯	◯	◯	◯	◯
public awareness/need	◯	◯	◯	◯	◯
staff buy-in or support	◯	◯	◯	◯	◯
sufficient staffing	◯	◯	◯	◯	◯
data systems/IT support	◯	◯	◯	◯	◯

Using home safety assessments and modification is more effective than our prior practices for reducing falls in older adults.

◯ Strongly Disagree ◯ Disagree ◯ Neutral ◯ Agree ◯ Strongly Agree

It is difficult to train staff to implement home safety assessments and modification.

◯ Strongly Disagree ◯ Disagree ◯ Neutral ◯ Agree ◯ Strongly Agree

Overall, I believe that it was complicated to implement home safety assessments and modification.

◯ Strongly Disagree ◯ Disagree ◯ Neutral ◯ Agree ◯ Strongly Agree

I believe that using home safety assessments and modification has required my organization to make substantial changes to our previous practice.

◯ Strongly Disagree ◯ Disagree ◯ Neutral ◯ Agree ◯ Strongly Agree

Home safety assessments and modification have required more work than can be done with current funding.

◯ Strongly Disagree ◯ Disagree ◯ Neutral ◯ Agree ◯ Strongly Agree

About you

What is your job title? __________________________________

What type of role to you play in the PWTF initiative (Please check all that apply.)

- ☐ Coordination/Management
- ☐ Administrative
- ☐ Community direct services, including community health workers
- ☐ Clinical services
- ☐ Referrals
- ☐ Consultation
- ☐ Leadership
- ☐ Quality improvement
- ☐ Community outreach

How many years have you worked at this organization? __________________________________

On average, how many hours per week do you work at this job? __________________________________

Age: __________________________________

Gender: __________________________________

Race/ethnicity (check all that apply):

- ☐ Black or African American
- ☐ Hispanic or Latino
- ☐ White
- ☐ Asian
- ☐ Native Hawaiian or Pacific Islander
- ☐ Other

If other, please specify: __________________________________

Education (highest level you have completed):

- ◯ Less than high school
- ◯ High school
- ◯ Some college/Associates
- ◯ College
- ◯ Graduate school or higher

Please indicate the language(s) you speak (check all that apply):

- ☐ English
- ☐ Spanish
- ☐ Portuguese
- ☐ Chinese
- ☐ Vietnamese
- ☐ Haitian Creole/French Creole
- ☐ Cambodian/Khmer
- ☐ Italian
- ☐ Russian
- ☐ French
- ☐ Arabic
- ☐ Other

Please share any final comments you have about the implementation of the PWTF initiative

____
