# Supplementary material for: A Mixed Methods Approach to Evaluate Partnerships and Implementation of the Massachusetts Prevention and Wellness Trust Fund
Source: Front Public Health. 2018 Jun 5;6:150. doi: 10.3389/fpubh.2018.00150 (PMC5996756; doi:10.3389/fpubh.2018.00150)
Supplement: Supplementary file 3 [file Data_Sheet_3.DOC]

Hello, my name is [name of interviewer].

**Purpose of Interview:**

The purpose of this interview is to better understand the implementation experience of your Prevention Wellness Trust Fund partnership. This includes the experience of planning, developing systems, and delivering interventions. We are also interested in understanding the barriers and facilitators to implementation your partnership has faced during the Prevention Wellness Trust Fund initiative with the intent to gather lessons learned and improve future work in this area. Throughout the intervention, I will refer to this work generally as the Prevention Wellness Trust Fund initiative. I will also ask about your work around specific health conditions (e.g. asthma or hypertension) and evidence-based intervention (e.g. Matter of Balance program to address elder falls or home visits to address asthma). *[You will refer to the sheet from Bekka on the conditions and interventions for each person here.]*

**Logistics and Confidentiality:**

In terms of logistics, this interview will be audio taped so that we have an accurate record of your thoughts. Please be assured that the tapes and your transcript will be kept confidential, so please answer openly and honestly. If, at any time, you feel that the questions are too sensitive, I would be happy to turn off the recorder during that portion of questioning. You may also skip any questions you wish during the interview. Within the next month, you will receive a $25 gift card via email as a token of appreciation for your time.

Do you have any questions for me? [Answer any questions]

Are you ready to begin? I’m going to start recording now.

**I. BACKGROUND** (10 Minutes)

**1. First, to help me understand who is involved with the Prevention Wellness Trust Fund initiative, will you please describe your title and role within your organization as it relates to the Prevention Wellness Trust Fund?** Background information

*PROBE:* Who do you report to?

**2. What other units/services WITHIN your organization do you work with to implement the Prevention Wellness Trust Fund initiative? How do you work together?** Networks & Communications – inside org

**3. What other organizations within your partnership do you work with to implement the Prevention Wellness Trust Fund initiative? How did you work together?**

Networks & Communications – inside partnership

**4. We are also interested in learning specifically about community-clinical partnerships. Is your organization involved in such a linkage?** Networks & Communications – inside partnership

*If yes: Can you describe one such partnership and tell us a bit about what has worked well and what hasn’t worked as well with that community-clinical partnership?*

**5. Prior to this initiative, what kind of strategies, if any, were you already using to address tobacco use/hypertension/asthma/elder falls [only ask for conditions assigned to this person]?** Relative Advantage

Walk through each PROBE below:

- *How long had you been implementing these strategies?*
- *How they are different from the PWTF strategies? Do you perceive the PWTF strategies to be a superior alternative? Why or Why not?*

**II. IMPLEMENTATION EXPERIENCE** (45 Minutes)

Now, I would like to hear about the process you have gone through to implement changes through the Prevention Wellness Trust Fund Project. When I refer to implementation I mean the process of putting to use or integrating evidence-based interventions within a community or clinical setting—this could include intervention delivery as well as developing supportive systems.

Today we’d like to focus on learning more about your experience implementing the [insert health conditions] interventions.

From my notes, it looks like you are involved in the implementation of [insert evidence-based interventions]. ***Refer to health conditions and evidence-based intervention list provided by Bekka.*** Is that correct?

Let’s start by talking about [insert evidence-based intervention].*Repeat 1&2 for each intervention they are involved with.*

1. **In general, can you explain how buy-in for this intervention occurred? Among your organization’s leadership? Among your staff?**

**2. Now, thinking about the whole process involved with implementing this evidence-based intervention, can you please tell me how it has been implemented in your partnership?**

Walk through each PROBE below:

- Who has led the changes in organization to implement PWTF activities?
- To what extent has your organization set goals for implementing the intervention? Have these changed over time? Process Goals
- What changes have you implemented?
- How complicated is the intervention? Complexity
- What have been the easiest parts of implementing this intervention?
- What have been the most challenging parts of implementing this intervention?
- Looking back, what do you think about the organizations involved with this intervention? Were the right number of organizations at the table? Who was missing? Who didn’t need to be on the project?
- How have clinical/community partnerships impacted implementation of this intervention?
- How have community health workers contributed to implement this intervention?
- How have you worked to address health equity to your implementation of these changes?
- Have you made any adaptations to this evidence-based intervention? What kind of changes did you make?
  - Can you tell me about the process you used to decide what adaptations to make?
  - How did you know if the adaptations were successful?

CLINICAL SITES ONLY:

- How have you changed your workflow to implement this intervention?
  - How were practice staff involved in developing the PWTF activities and workflow?
  - Were patients involved in developing the PWTF activities and workflow? If so, how?
- Did you plan or implement any performance feedback for clinicians on PWTF implementation?
  - If yes, how did feedback affect PWTF implementation?

**3. In thinking about the strategies used to address [tobacco/hypertension/asthma/elder falls], what has not been accomplished that was part of the plan? What happened?**

Now, I am interested in learning more about your thoughts on broad factors (both positive and negative) that may have affected your ability to implement evidence-based interventions through the Prevention Wellness Trust Fund project.

**4. Very broadly, can you describe the major factors that you think have influenced your ability to implement the evidence-based interventions for [tobacco/hypertension/asthma/elder falls]?**

*Ask the leading question and then walk through each PROBE category below:*

- Characteristics of the intervention/approach (such as the compatibility of the DPH-led TA and action planning with the way you do work in your organization and the complexity of the evidence-based strategy)
- Inner setting/Organizational infrastructure (e.g., available resources, data systems/IT support, sufficient staffing, size, physical layout, internal policies, leadership)
- Characteristics of individuals within the organization (e.g., role and experience of those involved, turnover)
- Influences outside of the organization (e.g., Local, state, or national policies; community characteristics; income, race, ethnicity of population served; public awareness/need).

**5. Have there been any competing priorities or other concurrent initiatives that influenced your ability to implement the PWTF interventions for [tobacco/hypertension/asthma/elder falls]?**

Optional PROBES:

- Examples might include PCMH certification, transition to ACO model, EHR changes, behavioral health integration efforts, other programming at YMCAs or elder centers
- Did other initiatives (such as PCMH or EHR modifications) help you to implement PWTF activities?
- Did you delay or decline to do other initiatives because of the PWTF? What did you delay or decline?

**For CLINICAL SITES ONLY** Quality of care

**6. How have the PWTF activities affected quality of care in your practice?**

Walk through each PROBE below:

- What intervention components were most important for improving quality of care?
  - Optional Probe: For example, electronic record system modifications, e-referrals, community partnerships, provider education
- What do you think could be done to alter the impact on quality of care?
- Are there any ways in which PWTF activities may have decreased quality of care?
  - Optional Probe: For example, provider time spent documenting results or discussing PWTF referral services that could have been spent counseling or on other health promoting activities.

**III. SUPPORT & ENGAGEMENT** (5-10 Minutes)

Now l want to hear briefly about any support and engagement you may have received along the way. I’m interested in understanding your broad impressions as well as those specific to [tobacco/hypertension/asthma/elder falls].

**1. What is your perception of the quality of the technical assistance and supporting materials of the Prevention Wellness Trust Fund evidence-based interventions?** Design Quality & Packaging

*Optional PROBES:*

- Learning collaboratives?
- TA calls/meetings with MDPH staff?
- Support from your coordinating partner?

**2. What level of involvement and support for the Prevention Wellness Trust Fund have you seen or heard from leaders within your organization during the implementation period?** Readiness for Implementation – Leadership Engagement

*Optional PROBES:*

- What are the roles of the leaders that have been most involved?
- How have they been involved or updated about the changes you planned/implemented?

**3. What kind of support do you have from others in your organization? Supervisor? Staff?** Implementation Climate

*Optional PROBE*

- How have staff gotten recognition for implementation of the PWTF interventions**?**

**4. Have any vocal staff, patients, elected officials, or community members emerged that either helped facilitate or inhibit this initiative? What happened?** Champions…Opinion Leaders

**IV. OPERATIONAL EXPERIENCES** (5-10 Minutes)

Now that we have discussed what you have been doing to address [tobacco/asthma/hypertension/elder falls] as part of the Prevention Wellness Trust Fund initiative, we want to know more about what it took to complete these changes.

**1. How would you gauge the time and effort required to implement the evidence-based interventions for [tobacco/asthma/hypertension/elder falls] over the course of the project?** Complexity

*PROBE:*

- Has additional or unforeseen time or effort incurred in planning/getting buy-in? implementing the evidence-based interventions on a day-to-day-basis? collecting data?

**2. What are the other resources required to implement the evidence-based interventions for [tobacco/asthma/hypertension/elder falls] over the course of the project?** Complexity & Readiness for implementation - Available Resources

*PROBE:*

- What resources would be needed to keep the interventions going on a long-term basis? IT support? Personnel time? Training? New purchases?

**V. PATIENT/COMMUNITY MEMBER EXPERIENCE** (10-15 minutes)

We are also interested in hearing about what you think about the experience your patients and community members have had with implementing the PWTF interventions for [tobacco/hypertension/asthma/elder falls]. *Ask only for primary condition.*

1. **What do patients/community members think of the evidence-based interventions for [tobacco/hypertension/asthma/elder falls] that were part of the PWTF initiative?** Patient Needs & Resources… Relative Advantage…Compatibility

*PROBES*

- What kind of impact did *PWTF* have on patients/community members?
  - Can you share a short story of someone you think was particularly positively impacted by PWTF?

**VI. REFLECTIONS ON INITIATIVE/SUSTAINABILITY** (5-10 Minutes)

Finally, we have a few questions to capture some of your overall reflections of this initiative.

**1. If you had the option, would you recommend continuing the Prevention Wellness Trust Fund initiative? Why or not?**

1. **What would it take to keep this initiative going in your community?**

Optional PROBES:

- Do you expect any of the partnerships to be useful for sustaining the work on the selected health conditions after the initiative is completed? Why or why not?
- What would make a specific partnership more likely to be sustained or utilized in the future? How does a history of working together (even before PWTF) come into play? What are other important factors?
- What would make a specific partnership less likely to be sustained after the funding ends?
- How easy would it be to undo the changes made thus far?

**3. Do you have any specific suggestions for other organizations that have not yet started implementing the evidence-based interventions that are part of the Prevention Wellness Trust Fund initiative?**

*Optional PROBE: on coalition building & evidence-based interventions*

Thank you again for your time today and for all the work you have done on behalf of the Prevention Wellness Trust Fund initiative. We look forward to hearing about continued work being done.

Notes on areas for specific intervention follow up, give quant surveys:

**Smoke-free environments**

- High Compatibility: “I think that promoting smoke-free environments to reduce tobacco use fits well with the way I like to work.”
- Low Int advantage: “Promoting smoke-free environments is more effective than our prior practices for reducing tobacco use”.

**Care management for high risk asthma patients**

- High Compatibility: “Using care management for high-risk asthma patients to reduce pediatric asthma is compatible with current activities in the organization.” And “I think that using care management for high-risk asthma patients to reduce pediatric asthma fits well with the way I like to work.”

**Headstart-asthma**

- Particularly low rankings
- Data
- Sufficient staffing
- Public awareness/need
- Equipment

**School-based asthma**

- Particularly low rankings
- Sufficient staffing!
- Data
